# Targeted neuronal reprogramming rescues memory and neural synchrony in Alzheimer’s disease

**DOI:** 10.1186/s43556-026-00481-w

**Published:** 2026-06-10

**Authors:** Marcos Galán-Ganga, Irene Rodríguez-Navarro, Sofía Zaballa, Alba Ramón-Lainez, Nerea Gómez-Rivada, Christian Peters, Joaquín Fernández-Irigoyen, Enrique Santamaría, M.Ángeles Rabadán, Jordi Alberch, Manuel J. Rodríguez, Daniel del Toro, Albert Giralt

**Affiliations:** 1 Departament de Biomedicina, Facultat de Medicina, Institut de Neurociències, Universitat de Barcelona, Barcelona, 08036 Spain; 2 Institut d’Investigacions Biomèdiques August Pi I Sunyer (IDIBAPS), Barcelona, 08036 Spain; 3 Centro de Investigación Biomédica en Red Sobre Enfermedades Neurodegenerativas (CIBERNED), Madrid, 28031 Spain; 4 Department of Molecules–Signaling–Development, Max-Planck Institute for Biological Intelligence, Martinsried, 82152 Germany; 5 Proteomics Platform, Navarrabiomed, Hospital Universitario de Navarra (HUN), Universidad Pública de Navarra UPNA, IdiSNA, Pamplona, 31008 Spain; 6 ZeClinics SL. and ZeNeuroid SL., Barcelona, Spain; 7 Production and Validation Centre of Advanced Therapies (Creatio), Faculty of Medicine and Health Science, University of Barcelona, Barcelona, 08036 Spain

**Keywords:** Dendritic spines, Yamanaka factors, Memory, Astrocytes, Microglia, PYK2

## Abstract

Alzheimer’s disease (AD) is the most prevalent neurodegenerative disorder and represents a major societal burden. Aging is the strongest risk factor for AD, and partial cellular reprogramming using Yamanaka factors (YFs) has recently emerged as a strategy to counteract age-associated dysfunction. However, the mechanisms by which partial reprogramming ameliorates AD-related phenotypes remain poorly defined. Here, we investigated whether targeted and intermittent expression of YFs in hippocampal neurons restores cognitive function and neural network integrity in the P301S mouse model of tauopathy. We first show that controlled YFs expression in hippocampal neurons increases excitatory synaptic transmission and enhances neural synchrony in GCaMP6-expressing neuronal networks. We then induced intermittent, neuron-specific YFs expression for six months in adult control and P301S mice. This intervention led to a sex-dependent improvement in cognitive and emotional behaviors in P301S mice, accompanied by a reduction in Tau pathology and partial restoration of epigenetic aging markers. At the molecular level, reprogramming restored the composition and signaling of N-methyl-D-aspartate receptor (NMDAR) macro-complexes, including key subunits and AD-associated risk factors such as proline-rich tyrosine kinase 2 (PYK2/PTK2B). Importantly, impaired hippocampal neural synchrony observed in P301S mice was also rescued. Together, these findings demonstrate that targeted, partial in vivo neuronal reprogramming reverses behavioral and network-level deficits in a mouse model of AD and identify NMDAR-associated signaling as a potential mechanistic mediator of this effect.

**Supplementary Information:**

The online version contains supplementary material available at 10.1186/s43556-026-00481-w.

## Introduction

Alzheimer’s disease (AD) is the most prevalent neurodegenerative disorder, accounting for 50%–70% of all dementia cases. In US, this proportion is expected to increase dramatically, with the number of affected individuals projected to rise from 6.5 million to over 15 million by 2050 [1]. AD primarily impairs cognitive domains such as memory, language, executive function, and visuospatial processing [2]. The hallmark proteins of AD, Amyloid-β (Aβ) and Tau, accumulate abnormally in the brain, exerting toxic effects that lead to progressive neuronal dysfunction and death [3]. The spatial distribution of Tau pathology closely correlates with the progression of clinical symptoms [4]. At the molecular level, AD is associated with a complex interplay of pathological processes including amyloid and Tau aggregation, inflammation, oxidative stress, glutamate excitotoxicity, synaptic dysfunction, and impaired autophagy [5]. Among these, one of the most critical and severely affected features is the loss of neural connectivity and synaptic plasticity [6, 7]. These deficits are strongly linked to cognitive decline and are driven by altered neural synchrony across different frequency bands [8, 9], as well as dysfunction in ionotropic glutamate receptors.

Among the ionotropic glutamate receptors N-methyl-D-aspartate receptors (NMDARs) are particularly affected in AD [10]. Genetic studies have identified single nucleotide polymorphisms in *GRIN2A* associated with phosphorylated Tau levels, and in *GRIN2B* with both phosphorylated Tau and Aβ1–42 levels [11, 12]. NMDAR activation triggers downstream signaling pathways, including the regulation of PYK2 and Glycogen synthase kinase 3 beta (or GSK3β) [13, 14]. GSK3β plays a key role in Tau hyperphosphorylation, a central process in AD pathology [15], while, PYK2 has emerged as a novel genetic risk factor for late-onset AD and interacts with Tau [16, 17]. Importantly, NMDAR-dependent signaling also regulates neural oscillations and synchrony [18].

Many of these pathological processes are closely linked to aging, the principal risk factor for AD [19, 20]. As such, the concept of accelerated aging has been proposed to better describe the progression of AD [21]. In this context, several anti-aging strategies have been proposed. Among the most promising is the use of Yamanaka factors (YFs), a set of four transcription factors (Oct4, Sox2, Klf4, and c-Myc, collectively known as OSKM) involved in epigenetic reprogramming and the induction of pluripotency [22–24]. Recent studies have demonstrated that tightly controlled OSKM expression in mice can enhance cognitive function and even reverse cognitive decline in AD models [25–29].

In the present study, we investigated the mechanisms by which transient YF expression modulates neuronal physiology and whether it could mitigate accelerated aging in AD. We first assessed the impact of YFs on neural network activity and electrophysiological properties in principal hippocampal neurons. We then used the P301S Tauopathy mouse model and restricted YF expression to mature hippocampal neurons. Our findings demonstrate that intermittent YF induction enhances neuronal synchrony, likely through modulation of NMDAR macro-complexes and downstream signaling pathways. These changes are associated with improved cognitive function and reduced neuropathology. Collectively, our results support the potential of transient YF-based reprogramming as a therapeutic strategy for Alzheimer’s disease.

## Results

### Intermittent YFs induction increases fire frequency and neural synchrony in hippocampal networks

**Fig. 1:**
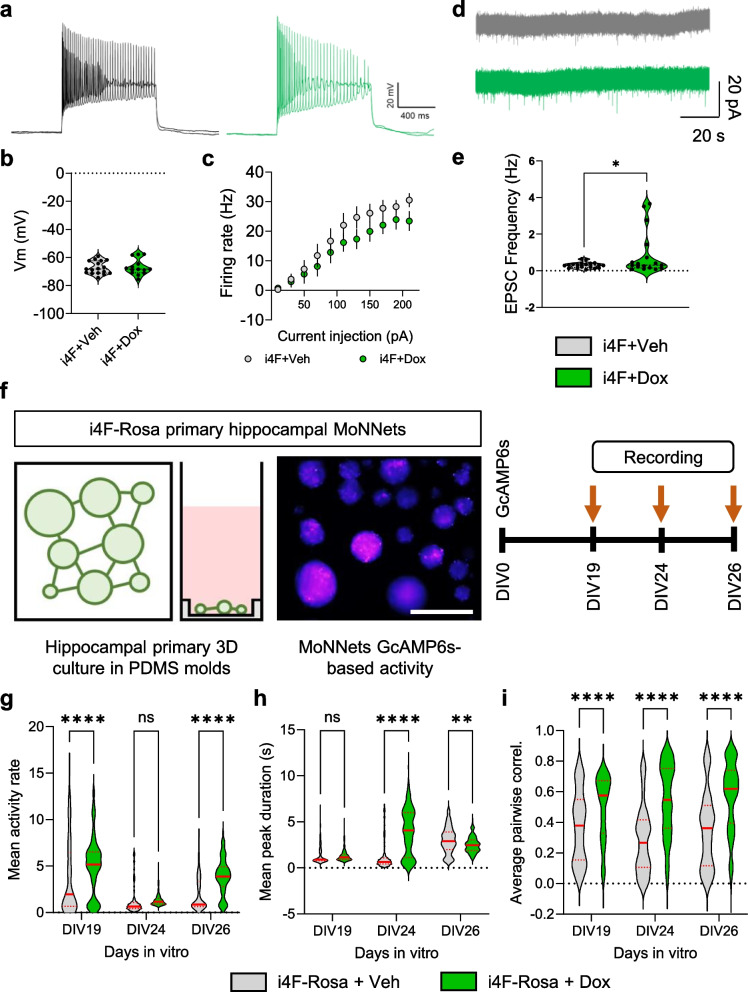
Effects of reprogramming in the physiology of hippocampal neurons. **a** Whole-cell current-clamp recordings of hippocampal pyramidal neurons in adult brain slices from i4F-A (or i4F) mice expressing the Yamanaka factors for 6 months (0.2 mg/mL doxycycline or Dox from 12-weeks-old to 36-weeks-old) or control (vehicle or Veh). **b** Membrane potential (Vm) for Veh and Dox expressing neurons. **c** Firing rates (in hertz) after injecting different current steps in Veh vs Dox. Veh *n* = 17 and Dox *n* = 15 neurons from 4 male mice/group. **d** Representative spontaneous excitatory postsynaptic current (sEPSC) recordings of hippocampal neurons expressing from the Dox group for 5 months or Veh brain slices. **e** Quantification of sEPSC frequency (Hz). (Unpaired t-test, t = 2,055, df = 34, *p = 0.047), Veh *n* = 19, Dox *n* = 17. **f** Primary hippocampal cells from i4F-Rosa mice were infected with AAV7m8.Syn.GCaMP6s.WPRE.SV40, and plated on a polydimethylsiloxane (PDMS) mold for self-organized assembly in neurospheres (or Modular Neuronal Network, a.k.a. MoNNets). Scale bar 250 µm, (n = 202–272 neurospheres). At day in vitro 14 (DIV14) and DIV16 MoNNets were treated with doxycycline (Dox, 1ug/ml) or vehicle (Veh). At DIV19, DIV24 and DIV26 MoNNets were monitored for Green fluorescent protein-Calmodulin-M13 protein, 6th generation, slow variant(GcAMP6s)-based activity. From the signal of MoNNets the following parameters were computed: (**g**) mean activity rate (two-way ANOVA, group effect: F_(1, 1359)_ = 60,5, *p* < 0.0001); (**h**) mean peak duration (two-way ANOVA, group effect: F_(1, 1342)_ = 76,22, *p* < 0.0001), and (**i**) the average pairwise Pearson correlation (two-way ANOVA, group effect: F_(1, 1342)_ = 259,8, *p* < 0.0001). Values are mean ± SEM. Bonferroni's multiple comparisons test was performed as a post hoc test

We first investigated whether partial reprogramming with YFs alters neuronal function. To this end, young i4F‑A (i4F) mice received hippocampal CA1 injections of pAAV8[TetOn]-TRE > ZsGreen1-rev(SYN1 > tTS:T2A:rtTA to drive rtTA expression (reverse tetracycline‑controlled transactivator). After 6 months of doxycycline treatment (0.2 mg/mL, 3 days/week in drinking water), brains were collected for ex vivo patch‑clamp recordings. The firing properties were measured after injecting different current steps (Fig. 1a). The membrane resting potential showed no differences between control and YF-expressing neurons (Fig. 1b). When measuring firing rates, the neurons shut down after higher current injections, likely due to depolarization block, with a similar increase in both groups (Fig. 1c). Additionally, we measured the spontaneous excitatory postsynaptic currents (sEPSCs), showing a significantly increased frequency for the reprogrammed i4F neurons (Fig. 1d, e). The latter could represent presynaptic changes, such as increased excitatory presynaptic neurotransmitter release or a higher number of functional excitatory synapses.

Since patch-clamp experiments showed only moderate effects following partial reprogramming, we next aimed to explore its impact in a more global, neural network–like model. For that we used the Modular Neuronal Network (MoNNets) approach [30] designed to recognize neural patterns (based on GcAMP6s-dependent calcium activity) in primary neurons organized in neurospheres (Fig. 1f). Thus, primary neurons from i4F-Rosa mice were treated with Dox at day in vitro 14 (DIV14) and DIV16 and GcAMP6s-dependent activity was monitored at DIV19, DIV24 and DIV26 (Fig. 1f). This reprogramming protocol globally increased the mean activity rate (Fig. 1g), the mean peak duration (Fig. 1h) and the average pair correlation or synchrony (Fig. 1i) of these MoNNets, with the increase in synchrony being the most clear and consistent. Altogether, these results may indicate that in vivo intermittent YF induction or reprogramming enhances neural synchrony and function.

### Intermittent YFs induction in adult hippocampal neurons improves the phenotype of the P301S transgenic mouse model of Alzheimer’s disease in a sex-dependent manner

**Fig. 2:**
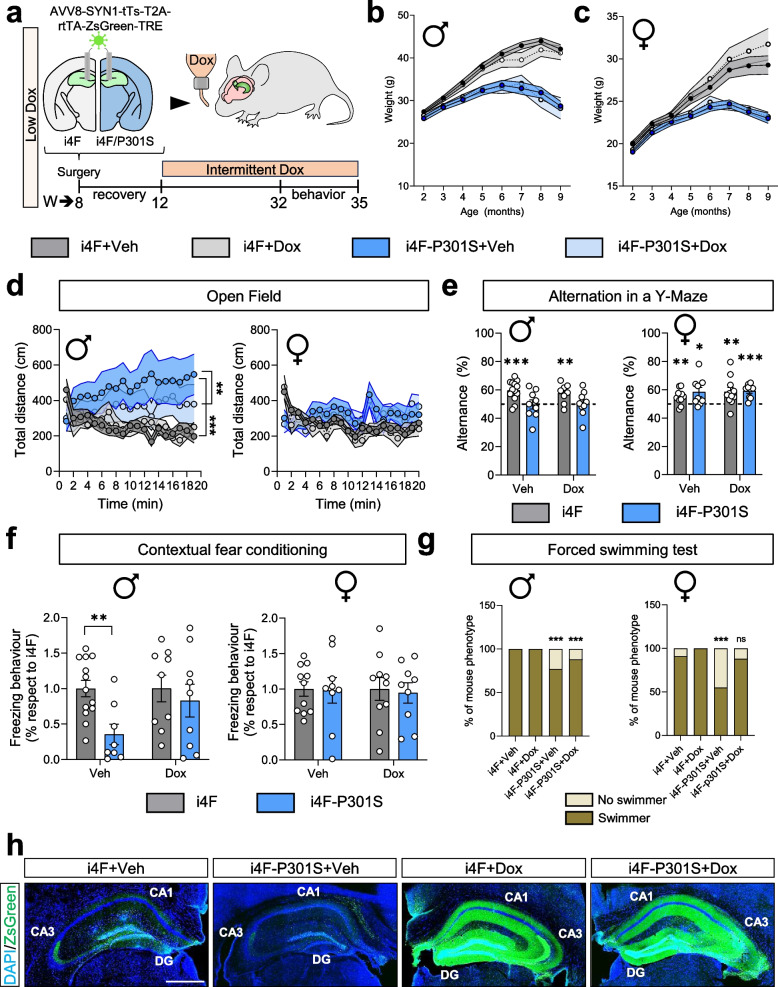
Behavioral effects of In vivo YF induction in the hippocampal principal neurons of the P301S mouse model. **a** Schematic and delivery of AAV8-SYN1-tTs-T2A-rtTA-ZsGreen-TRE into the hippocampus of the four groups of mice (i4F + Veh, i4F + Dox, i4F-P301S + Veh and i4F-P301S + Dox). Dox treatment lasted from 12-weeks-old to 36-weeks-old. Males and females were studied. Body weight from all groups in both males (**b**) two-way ANOVA group effect, F_(3, 532)_ = 90,34, *p* < 0.0001) and females (**c**) two-way ANOVA group effect, F_(3, 636)_ = 56,59, *p* < 0.0001) during the entire experimental procedure. **d** Locomotor activity and exploration in the open field test in both males (two-way ANOVA group effect, F_(3, 703)_ = 36,83, *p* < 0.0001) and females (two-way ANOVA group effect, F_(3, 35)_ = 1,283, *p* = 0.29). **e** Quantification of spatial spontaneous alternation in the Y-maze in both males (two-way ANOVA group effect, F_(1, 35)_ = 10,82, *p* = 0.0023) and females (two-way ANOVA group effect, F_(1, 36)_ = 0,62, *p* = 0.434). **f** Percent time spent with respect to i4F in freezing behavior in the fear conditioning paradigm in both males (two-way ANOVA interaction effect, F_(1, 35)_ = 5,173, *p* = 0.0292) and females (two-way ANOVA interaction effect, F_(1, 35)_ = 0,013, *p* = 0.908). Values are relativized to control groups. **g** Percentage of mice successfully performing the forced swimming test in both males (Fisher’s exact test, compared with i4F + Veh, *p* < 0.0001) and females (Fisher’s exact test, compared with i4F + Veh, *p* < 0.0001). **h** ZsGreen (ZG) immunoreactivity in dorsal hippocampus 3 weeks after viral injection. Scale bars, 400 μm. Values are mean ± SEM. Bonferroni’s multiple comparisons test was performed as a post hoc test. In all tests, males, n = 8–13 mice/group; females, n = 9–11 mice/group. Validation of rtTA-dependent ZsGreen (in green) signal co-labeled with DAPI (blue) after 5 months’ Dox and Veh intermittent treatment in all groups of mice. *Cornu Ammonis* area 1 and 3 (CA1, CA3); Dentate gyrus (DG); Stratum Oriens (SO); *Stratum Pyramidale* (SP); *Stratum Radiatum* (SR). Scale bar: 500 microns

Our initial experiments in brain slices and in in vitro neural networks indicated that intermittent and partial reprogramming induces enhancing effects in neural connectivity and synchrony. Interestingly, Alzheimer’s disease (AD), and concretely tauopathy, is commonly associated with synaptic loss and altered connectivity, both of which are considered major contributors to cognitive decline [31]. Thus, we decided to test whether this approach could improve the cognitive deficits observed in the P301S transgenic mouse model of AD. We previously demonstrated that transient YF induction is well tolerated in adult neurons, without affecting identity, function, or viability [25]. Based on these findings, we established an in vivo intermittent YF induction protocol in terms of dosage and experimental design [25]. P301S mice were crossed with the i4F‑A (i4F) line to generate i4F‑P301S mice along with littermate controls (i4F). This double‑mutant line was indistinguishable from the parental strains regarding viability (Fig. S1). YF expression was driven by an adeno‑associated virus encoding SYN1‑dependent rtTA, injected into the dorsal hippocampus of 8‑week‑old mice (Fig. 2a). From 12 to 36 weeks of age, both groups (i4F‑P301S and i4F controls) received either vehicle or doxycycline (0.2 mg/mL), administered 3 days on and 4 days off per week. Importantly, prolonged Dox treatment did not affect the parameters analyzed, as previously reported [25]. At 8 months, all groups underwent extensive behavioral and histological analyses. (Fig. 2a). We first evaluated behavioral phenotypes in our four groups of male and female mice (i4F + Veh, i4F + Dox, i4F-P301S + Veh and i4F-P301S + Dox).

We first monitored body weight across all groups and sexes. A progressive reduction in body weight was observed in i4F‑P301S mice of both sexes (males: Fig. 2b; females: Fig. 2c), with no effect of Dox treatment. We then assessed locomotor and exploratory behavior using the open field test. Male i4F‑P301S + Veh mice exhibited increased hyperlocomotion compared to i4F + Veh controls, which was significantly normalized in i4F‑P301S mice treated with Dox (Fig. 2d). Interestingly, hyperlocomotion has previously been reported in P301S mice [32, 33] and is consistent with the hyperactivity observed in AD patients [34]. In contrast, we did not detect changes in female groups (Fig. 2d). Next, the four groups of mice performed the Y-maze in which we evaluated their spatial working memory skills. In this test both male i4F-P301S + Veh and i4F-P301S + Dox mice showed a strongly reductions in their percentage of alternation that was not affected by reprogramming (Fig. 2e). In females, all groups showed equal rates of spatial working memory skills (Fig. 2e). Mice were next subjected to the contextual fear conditioning, and again, i4F-P301S + Veh males displayed lesser rates of freezing behavior, a phenotype that was recovered in i4F-P301S + Dox mice (Fig. 2f). In the case of females, all groups showed similar rates of freezing behavior in the contextual fear conditioning (Fig. 2f). The last behavioral paradigm was the forced swimming test, used to evaluate potential changes in emotional and depressive-like behaviors. In this paradigm, both i4F-P301S + Veh and i4F-P301S + Dox male mice showed increased and similar percentages of individuals incapable of floating and struggling (Fig. 2g). In contrast, this inability to float was even more pronounced in i4F-P301S + Veh female mice and completely rescued in i4F-P301S + Dox females (Fig. 2g). Finally, we included in the AAVs a ZsGreen reporter driven by a tetracycline operator (TRE) promoter to visualize and confirm the presence of neurons expressing YFs after Dox treatment (Fig. 2h). In addition, we confirmed the increased expression of Klf4 (one of the YFs) in the CA1 area, specifically in i4F + Dox and i4F-P301S + Dox male and female mice (Fig. S2). We also confirmed that the viral transduction and YFs expression occurred specifically in the hippocampus of i4F + Dox and i4F-P301S + Dox mice (Fig. S3). In this line, YFs were expressed, as expected, in principal hippocampal neurons without affecting their neuronal identity or viability (Fig. S3). Finally, we verified neuronal specificity of our reprogramming and YFs expression (Fig. S4). In conclusion, partial reprogramming improved crucial cognitive and behavioral phenotypes, mostly in males but also in females.

### Intermittent YFs induction in adult hippocampal principal neurons reduces Tauopathy and restores epigenetic signatures and neuroinflammatory processes in the P301S mice

**Fig. 3:**
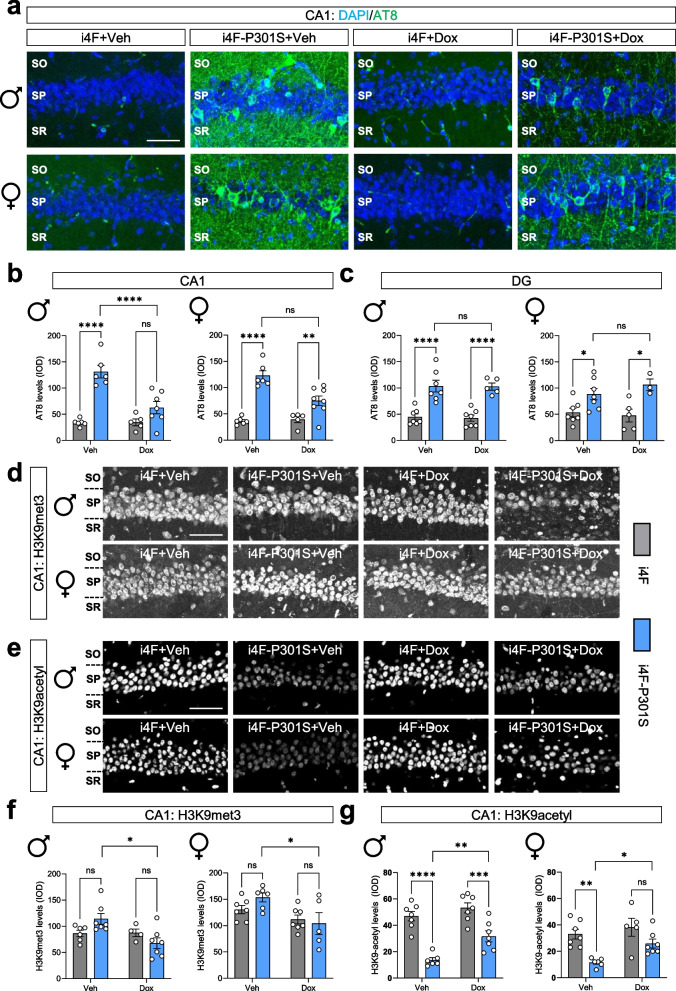
Evaluation of Tau pathology and epigenetic markers in brains of reprogrammed mice. **a** AT8 staining for phosphorylated human Tau (at pS202/pT205 residues, green) co-labeled with DAPI (blue) in the hippocampal *Cornu Ammonis* area 1 (CA1) of i4F + Veh, i4F + Dox, i4F-P301S + Veh and i4F-P301S + Dox mice. Scale bar: 50 microns. **b** Quantification of AT8 levels (integrated optical density or IOD) in dorsal CA1 in both, males (two-way ANOVA group effect, F_(1, 20)_ = 42,67, *p* < 0.0001) and females (two-way ANOVA group effect, F_(1, 21)_ = 58,10, *p* < 0.0001) groups. **c** Quantification of AT8 levels (IOD) in dorsal DG in both, males (two-way ANOVA group effect, F_(1, 22)_ = 55,05, *p* < 0.0001) and females (two-way ANOVA group effect, F_(1, 18)_ = 16,67, *p* < 0.0001). Tri-methylated H3K9 **d** and acetylated H3K9 (**e**) labeling in the hippocampal CA1 of the same four groups of mice. Scale bars: 50 microns. **f** Quantification of Tri-methylated H3K9 levels (IOD) in dorsal CA1 in both, males (two-way ANOVA interaction effect, F_(1, 20)_ = 6,097, *p* = 0.0227) and females (two-way ANOVA treatment effect, F_(1, 21)_ = 9,309, *p* = 0.0061) groups. **g** Quantification of acetylated H3K9 levels (IOD) in dorsal CA1 in both, males (two-way ANOVA group effect, F_(1, 23)_ = 60,00, *p* < 0.0001) and females (two-way ANOVA group effect, F_(1, 21)_ = 18,37, *p* = 0.0003) groups. Values are mean ± SEM. Males, n = 4–7 mice/group; females, n = 5–7 mice/group

To test whether intermittent reprogramming modulated Tau pathology in 9-month-old i4F-P301S mice, an immunostaining against AT8, which recognizes abnormally phosphorylated Tau [35], was performed. We then determined the intensity of mutant and hyperphosphorylated Tau in the hippocampal CA1 and DG (Dentate Gyrus) of i4F-P301S + Dox and i4F-P301S + Veh mice (Fig. 3a). As expected, aberrantly hyperphosphorylated Tau in the hippocampal CA1 and DG in all i4F-P301S groups of mice was significantly increased when compared with their respective control i4F groups (Fig. 3a, c). However, i4F-P301S + Dox male mice displayed significantly reduced levels of aberrant AT8-positive intensity in CA1 when compared with i4F-P301S + Veh male mice (Fig. 3a, b). Unfortunately, this reduction was not significant in i4F-P301S + Dox female mice in comparison with i4F-P301S + Veh female mice (Fig. 3a, b). Regarding to the DG, no ameliorations of AT8-positive intensity were observed in any i4F-P301S group (Fig. 3a, c). Since reprogramming and YFs induction provoke substantial changes in epigenetic markers [36], we moved to evaluate the state of H3K9met3 and H3K9acetyl epigenetic markers, which are altered in P301S mice [37–39]. We focused on the CA1 region, where AT8 signal amelioration was observed (Fig. 3d, g). First, we observed a global trend to increased H3K9met3 levels in both male and female i4F-P301S + Veh mice, which were significantly reduced and normalized to control levels in i4F-P301S + Dox male and female mice (Fig. 3d, f). In contrast, H3K9acetyl levels were markedly reduced in i4F-P301S + Veh mice of both sexes compared with their respective i4F + Veh controls and were significantly, though not completely, restored in i4F-P301S + Dox mice (Fig. 3e, g). Altogether, these neuropathological results indicate that intermittent reprogramming reduced aberrant hyperphosphorylated Tau aggregates specifically in CA1 in the P301S mice, accompanied by a recovery and normalization of altered levels of H3K9met3 and H3K9Acetyl epigenetic markers.

**Fig. 4:**
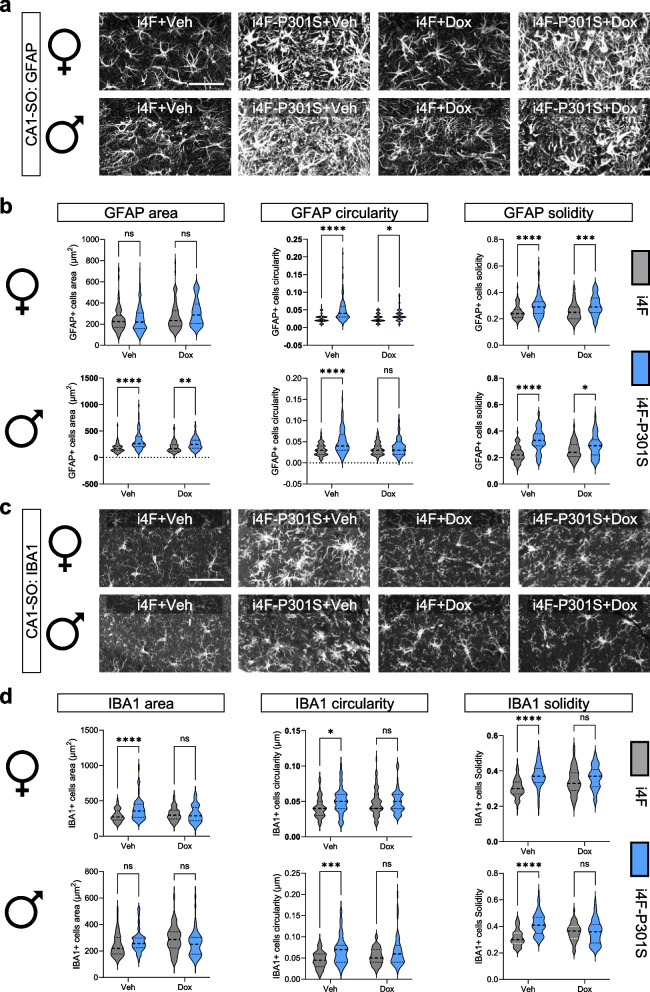
Morphological and neuroinflammatory profiles in brains of reprogrammed mice. **a** Glial fibrillary acidic protein (GFAP) staining for astrocytes in the hippocampal *stratum radiatum* of the CA1 of male and female i4F + Veh, i4F + Dox, i4F-P301S + Veh and i4F-P301S + Dox mice. Scale bar: 80 microns. **b** GFAP-positive cells were evaluated in females (upper row) in terms of cell area (left panel, two-way ANOVA, group effect: F_(1, 341)_ = 1,775, *p* = 0.183), cell circularity (middle panel, two-way ANOVA, group effect: F_(1, 339)_ = 44,00, *p* < 0.0001) and cell solidity (right panel, two-way ANOVA, group effect: F_(1, 341)_ = 31,64, *p* < 0.0001). n = 60–119 cells/group (6–7 mice per condition were used). GFAP-positive cells were evaluated in males (lower row) in terms of cell area (left panel, two-way ANOVA, group effect: F_(1, 299)_ = 46,67, *p* < 0.0001), cell circularity (middle panel, two-way ANOVA, group effect: F_(1, 297)_ = 22,61, *p* < 0.0001) and cell solidity (right panel, two-way ANOVA, group effect: F_(1, 299)_ = 59,52, p < 0.0001). n = 61–89 cells/group (6–7 mice per condition were used). **c** Ionized calcium-binding adapter molecule 1 (IBA1) staining for astrocytes in the hippocampal *stratum radiatum* of the CA1 of male and female of the same four groups of mice. Scale bar: 80 microns. **d** IBA1-positive cells were evaluated in females (upper row) in terms of cell area (left panel, two-way ANOVA, group effect: F_(1, 243)_ = 9,909, *p* = 0.0019), cell circularity (middle panel, two-way ANOVA, group effect: F_(1, 243)_ = 6,472, *p* = 0.016) and cell solidity (right panel, two-way ANOVA, group effect: F_(1, 243)_ = 27,98, *p* < 0.0001). Values are mean ± SEM. n = 45–78 cells/group (6–7 mice per condition were used). IBA1-positive cells were evaluated in males (lower row) in terms of cell area (left panel, two-way ANOVA, group effect: F_(1, 213)_ = 0,05258, *p* = 0.818), cell circularity (middle panel, two-way ANOVA, group effect: F_(1, 213)_ = 18,48, *p* < 0.0001) and cell solidity (right panel, two-way ANOVA, group effect: F_(1, 213)_ = 22,66, *p* < 0.0001). n = 44–62 cells/group (6–7 mice per condition were used). Values are mean ± SEM. Bonferroni’s test was used as a post hoc in all the analyses

We wondered if the beneficial effects at behavioral and neuropathological levels mediated by reprogramming could be linked to a general and indirect effect in neuroinflammation. To address this, we evaluated the area, circularity and solidity of GFAP and IBA1-positive cells in the *stratum radiatum* of the CA1 of the hippocampus using immunofluorescence. First, we observed that circularity and solidity scores in GFAP-positive astrocytes equally increased in both female i4F-P301S + Veh and i4F-P301S + Dox mice (Fig. 4a, b). Similarly, the area and solidity of GFAP-positive astrocytes equally increased in both, male i4F-P301S + Veh and i4F-P301S + Dox mice (Fig. 4a, b). However, a subtle but significant rescue in GFAP-positive cells circularity was observed in P301S + Dox mice compared with i4F-P301S + Veh mice (Fig. 4a, b). Next, we evaluated the same parameters in IBA1-positive microglia. Interestingly, IBA1-positive cells area, circularity and solidity were all increased in i4F-P301S + Veh female mice but rescued in i4F-P301S + Dox female mice (Fig. 4c, d). Likewise, circularity and solidity scores were increased in i4F-P301S + Veh males but rescued in i4F-P301S + Dox male mice (Fig. 4c, d). We further explored the functional state of microglia and astroglia. First, we observed that TNFα, a pro-inflammatory molecule mostly produced by astrocytes [40], was up-regulated in the hippocampus of i4F-P301S + Veh but fully recovered i4F-P301S + Dox mice (Fig. S5a-b). Similarly, CD68-labeled phagocytosis activity [41] was also assessed in hippocampal microglia and we found it increased in P301S + Veh but completely rescued i4F-P301S + Dox mice (Fig. S5c-d). Finally, looking at our mass spectrometry study (Supplementary Table 1, see below), we also observed that some disease associated astroglia (DAA) proteins (such as SLC17A7, SLC17A6) were altered in P301S + Veh but improved i4F-P301S + Dox mice (Fig. S5e). These results indicate that targeted intermittent reprogramming was able to improve several dysfunctional states in microglia and astrocytes in the hippocampus of these mice.

### Molecular signatures of reprogrammed P301S hippocampi suggest a major effect in N-Methyl-D-Aspartate receptors macro-complexes

**Fig. 5:**
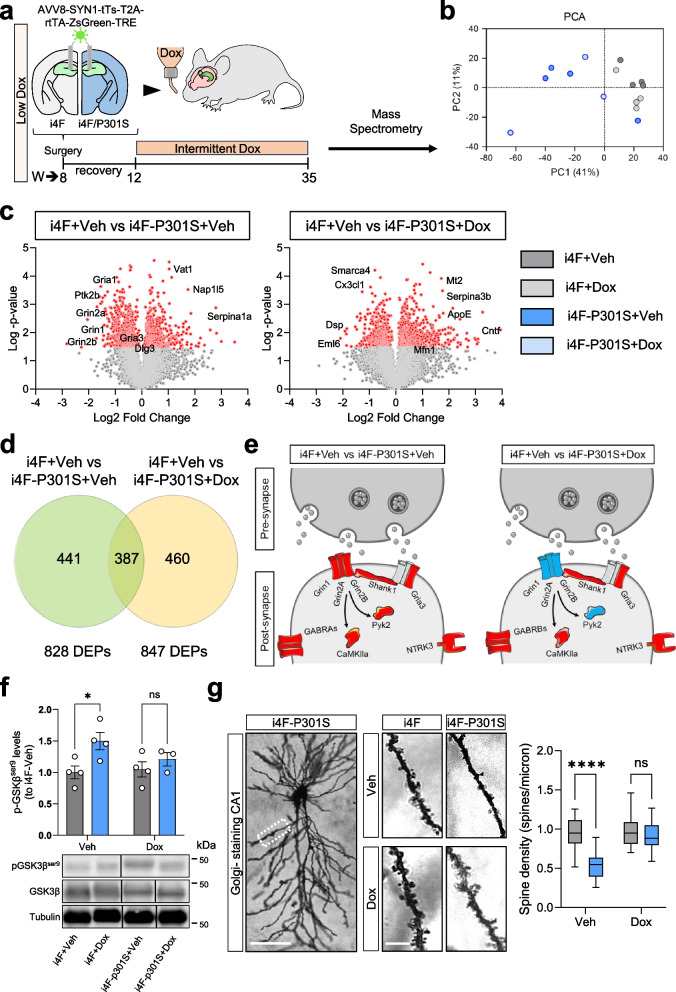
Proteomic signatures and structural synaptic plasticity in the hippocampus of reprogrammed P301S mice. **a** Schematic representation in a new cohort of mice (n = 3–4/group; only males) showing the hippocampal infection with AAV8-SYN1-tTs-T2A-rtTA-ZsGreen-TRE construct with i4F and i4F-P301S; the drawing also shows the timing of intermittent Dox treatment (from 12-weeks-old to 36-weeks-old). **b** Principal component analysis (PCA) scores plot of liquid chromatography-mass spectrometry (LC–MS) data from i4F + Veh, i4F + Dox, i4F-P301S + Veh and i4F-P301S + Dox mouse hippocampi. **c** Volcano plots showing differentially expressed proteins (DEPs) when comparing i4F + Veh vs i4F-P301S + Veh (left panel) and i4F + Veh vs i4F-P301S + Dox (right panel). **d** Venn diagram showing the total number of DEPs in both, i4F-P301S + Veh and i4F-P301S + Dox mice compared with i4F + Veh mice. **e** Schematic representation of excitatory synaptic proteins differentially expressed in i4F-P301S + Veh mice (left synapse) and i4F-P301S + Dox mice (right synapse) when compared with i4F + Veh mice. KEGG 2021, SynGo 2024 and Panther2016 databases were used. Proteins depicted in red are down-regulated and proteins in blue are not significantly different. **f** Densitometric quantification of hippocampal phosphorylated GSK3β^ser9^ levels in i4F + Veh, i4F + Dox, i4F-P301S + Veh and i4F-P301S + Dox mice (two-way ANOVA, group effect: F_(1, 11)_ = 7,429, *p* = 0.019). **g** Golgi-Cox-stained dendrites from dorsal CA1 pyramidal neurons (secondary apical dendrites). Scale bars, 40 μm (left panel) and 7 μm (middle panels). Quantification of spine density in dendrites as in middle panel (two-way ANOVA, group effect: F_(1, 192)_ = 87,51, *p* < 0.0001; 49 dendrites per group/6–7 male mice were used per condition). Values are mean ± SEM. Bonferroni’s test was used as a post hoc in all the analyses

We then sought to identify potential molecular mechanisms underlying the phenotypic improvements observed in the i4F-P301S + Dox mice compared with i4F-P301S + Veh mice. We repeated the entire protocol, omitting behavioral phenotyping to avoid nonspecific effects of long-term mice manipulation on subsequent molecular studies (Fig. 5a). Thus, we subjected the hippocampi of reprogrammed mice to mass spectrometry (Table S1). Here we focused on males, as they showed better global phenotype improvements. First, principal component analysis (PCA) of the proteomic profiles (detecting up to ~5000 proteins) clearly separated i4F and P301S groups, with several i4F-P301S + Dox samples located between i4F + Veh and P301S + Veh mice (Fig. 5b). The latter result suggests a partial recovery of the molecular profile in i4F-P301S + Dox relative to i4F-P301S + Veh. We then focused on the differentially expressed proteins (DEPs). Volcano plots of the total DEPs indicated that many proteins were up- and down-regulated in i4F-P301S + Veh and i4F-P301S + Dox mice compared with i4F + Veh controls (Fig. 5c). We noticed that several of these proteins were synaptic such as scaffold proteins (SAP102 or *Dlg3*), ionotropic receptors subunits (GluA3, GluN1) and synaptic plasticity kinases (CaMKIIα or *Camk2a* and PYK2 or *Ptk2b*) (Table S1 and Fig. S6). We also observed that 828 DEPs were identified in the hippocampus of i4F-P301S + Veh mice compared with i4F + Veh mice, whereas 847 DEPs were identified in the i4F-P301S + Dox mice when compared with i4F + Veh mice (Fig. 5d). The latter result indicates that neuronal partial reprogramming induces a lot of de novo molecular changes that could account for the phenotype improvements identified in i4F-P301S + Dox mice. We deepen on the ontologies and major pathways affected by reprogramming using well-established databases (EnrichR, Panther and SynGO). Focusing on synaptic changes, we found that, among the several molecular changes that were observed in i4F-P301S + Veh mice, the N-methyl-D-aspartate receptors (NMDARs) macro-complexes were completely rescued in the i4F-P301S + Dox mice (Fig. 5e and Fig. S6). This NMDAR macro-complexes included GluN1 (*Grin1*), GluN2A (*Grin2a*), GluN2B (*Grin2b*) and PYK2 (*Ptk2b*) but not SHANK1 (*Shank1*) or CaMKIIα (Fig. 5e and Fig. S6). Since the NMDAR macro-complexes can signal downstream trough PYK2 [42] to activate GSK3β [43, 44], which in turn phosphorylates Tau and induces its accumulation of neurofibrillary tangles [45], we evaluated the activation state of GSK3β in the same samples. We observed that GSK3β was hyperphosphorylated in i4F-P301S + Veh mice as described elsewhere [46] but completely recovered in i4F-P301S + Dox mice (Fig. 5f). Finally, we aimed to relate these molecular changes to structural synaptic plasticity. For this purpose, we quantified spine density in apical dendrites of CA1 hippocampal neurons across all experimental groups (Fig. 5g). Excitatory spine density was significantly reduced in pyramidal neurons of i4F-P301S + Veh mice but completely rescued in pyramidal neurons of i4F-P301S + Dox mice (Fig. 5g). Altogether, these findings suggest that the improved phenotypes observed in i4F-P301S + Dox mice could be due to a restoration of the NMDAR-PYK2-GSK3β-Tau pathway although further experimentation should be performed to deep into this potential molecular mechanism.

### Hippocampal synchrony but not long-term potentiation is restored by intermittent YF induction of principal neurons in P301S mice

**Fig. 6:**
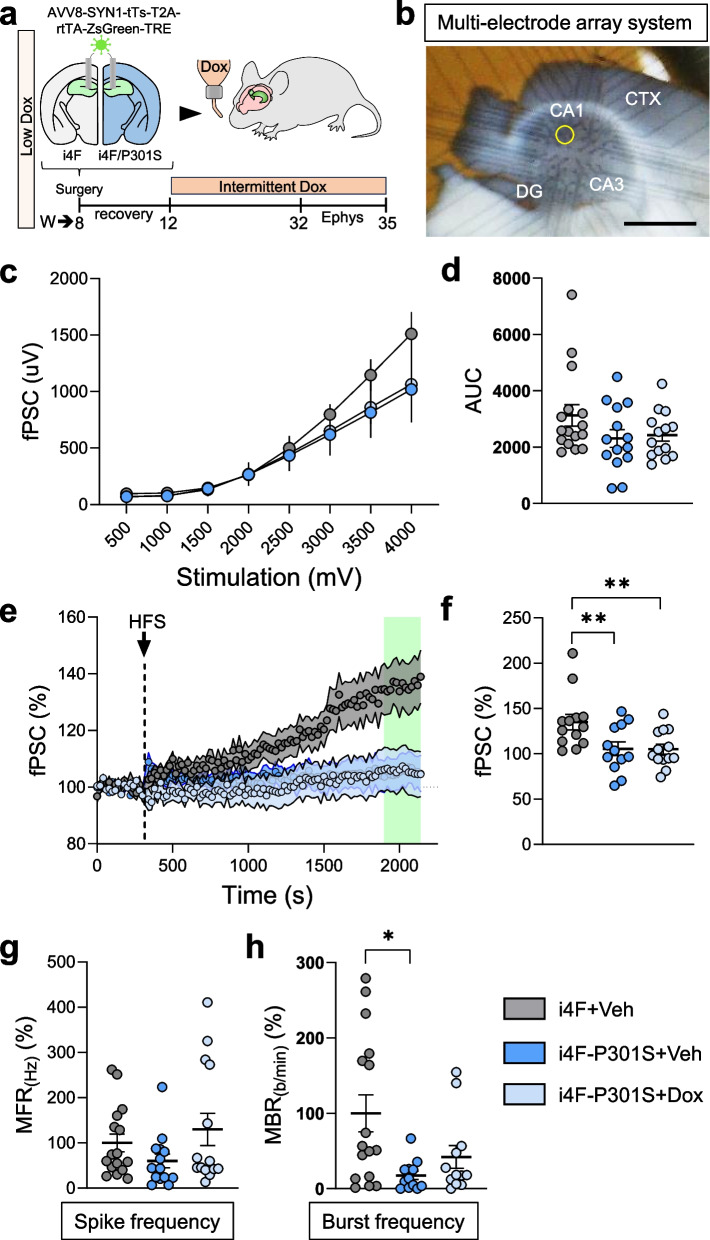
Electrophysiological characterization of reprogrammed P301S mice. **a** Schematic representation showing the hippocampal infection with AAV8-SYN1-tTs-T2A-rtTA-ZsGreen-TRE construct with i4F and i4F/P301S; the drawing also shows the timing of intermittent Dox treatment (from 12-weeks-old to 36-weeks-old). **b** Representative image of a mouse brain transversal section showing the location of stimulation electrode for hippocampal field post-synaptic currents (fPSC) recordings. Scale bar, 1000 μm. **c** The amplitude of electrical intensity-induced hippocampal fPSC in the 3 groups in the input–output study. **d** Boxplot shows the area under the curve (AUC) quantification of the input–output curves. **e** The graph shows the time course and quantification of fPSC evoked at hippocampal CA1 synapses and the LTP induced after a high frequency stimulation (HFS). **f** The boxplot shows the mean amplitude of fPSC evoked at 25–30 min after HFS. fPSC amplitudes are represented as a percentage of baseline. Boxplots in (**g**) and (**f**) show quantification of neuronal spontaneous activity in the hippocampal CA1 in terms of (**g**) mean spike frequency (MFR) and (**f**) mean burst frequency (MBF) of the three groups. Frequencies are represented as a percentage of activity respect to the i4F + Veh group. Data represent mean ± SEM and differences were analyzed by one-way or two-way ANOVA with Bonferroni post-hoc comparisons. * *p* < 0.05; ** *p* < 0.01. Each point represents data from an individual slice. n = 16 slices per condition from 22 mice (3–4 female and 3–4 male per condition). Some data points were discarded after outlier analysis

The recovery of several cognitive skills in i4F-P301S + Dox mice, concomitant with the restoration of the NMDAR-PYK2-GSK3β-Tau pathway and hippocampal spine density, motivated us to further investigate the underlying physiological changes. Since we initially observed that YFs expression enhances synaptic firing and synchrony in neural networks, we next sought to evaluate several electrophysiological parameters in reprogrammed mice. Thus, we repeated the entire protocol without behavioral phenotyping to avoid nonspecific effects of long-term mice manipulation in the ulterior electrophysiological studies (Fig. 6a). On the last day of Dox treatment, brains were collected and subjected to multi-electrode arrays recordings (MEAs, Fig. 6b).

We recorded field potential synaptic currents (fPSCs) in the CA1 region following stimulation of CA3 afferents at increasing intensities to generate a normalized input–output curve. We calculated the area under the curve (AUC) to assess basal synaptic efficacy and excitability between experimental groups. Stimulation of CA3 evoked progressively larger fPSCs in the CA1 of all slices (Fig. 6c; stimulation effect: F_(7, 287)_ = 163.1, *p* < 0.001; two-way ANOVA) without differences between groups (group effect: F_(2,41)_ = 2.1, *p* = 0.133; two-way ANOVA). AUC quantification confirmed the absence of significant effects between groups (Fig. 6d; F_(2,18)_ = 0.0253, *p* = 0.779; one-way ANOVA), indicating no alterations in basal synaptic efficacy or excitability in any group of mice. We next evaluated the effect of reprogramming on synaptic plasticity in CA1 neurons by inducing long-term potentiation (LTP) through high frequency stimulation (HFS) of CA3 afferents. We recorded fPSCs following electrical stimulation set to evoke 40% of the maximal response identified in the input–output assay. Under these conditions, only i4F + Veh slices showed a significant increase in fPSC amplitude (35.2%), indicating successful LTP induction (Fig. 6e). This potentiation was abolished in both, i4F-P301S + Veh and i4F-P301S + Dox slices (Fig. 6f; F_(2,36)_ = 5.530, *p* = 0.008; one-way ANOVA). These results indicate a global LTP impairment, a key form of synaptic plasticity, in P301S mice that is not rescued by reprogramming.

We then assessed whether YFs intermittent expression modifies the hippocampal functional excitability in brain slices of i4F + Veh, i4F-P301S + Veh and i4F-P301S + Dox mice. To this end, we measured neuronal spike rate and bursting activity in the pyramidal CA1 area. We observed no significant effects between groups on the overall spike rating (Fig. 6g; F_(2, 41)_ = 1.932, *p* = 0.158; one-way ANOVA). However, the spike-train analysis revealed a reduction in burst rate induced in i4F-P301S + Veh slices, which was rescued in i4F-P301S + Dox slices when compared to i4F + Veh slices (Fig. 6h, F_(2,37)_ = 5.268, *p* = 0.0097). These results suggest that temporal organization of spontaneous neuronal firing in CA1 is impaired in i4F-P301S + Veh mice, potentially reflecting subtle alterations in network excitability or synaptic integration, which can be partially ameliorated by reprogramming.

## Discussion

Here we demonstrate that intermittent partial reprogramming inducing conditional expression of the Yamanaka factors (YFs) in hippocampal neurons enhances neuronal activity, connectivity, and synchrony in neural network models. Reprogramming hippocampal principal neurons in P301S mice led to a rescue of several cognitive deficits. Notably, we also observed concurrent changes in Tau-related and neuroinflammatory pathologies, epigenetic signatures, and neural synchrony, consistent with our in vitro findings. Subsequent proteomic and biochemical analyses suggest that these improvements are likely driven by a potential restoration of the NMDAR–PYK2–GSK3β–Tau signaling pathway.

Here we demonstrate a broad and substantial recovery of multiple cognitive functions in the P301S model of AD by partial intermittent reprogramming. We observed improvements in hippocampal-dependent memory, reduced locomotor agitation, and alleviation of behavioral despair, all well-established deficits in this model [33]. Intriguingly, we also show for the first time that partial reprogramming via intermittent YFs induction exerts its effects in a sex-dependent manner. In general, P301S females exhibited milder behavioral phenotypes, consistent with previous reports [47]. Likewise, aberrant Tau hyperphosphorylation was more effectively rescued in male than in female P301S mice. Regarding on potential molecular explanations for these sex differences it is noteworthy that the P301S human 1N4R Tau transgene is inserted on chromosome 3, ruling out sex-linked inheritance as a contributing factor [48]. Finally, we observed that both sexes showed comparable changes in epigenetic signatures and neuroinflammatory profiles, including the recovery of associated parameters. Taken together, these findings suggest that while both the P301S mutation and intermittent reprogramming exert common effects across sexes, they also produce sex-specific outcomes in certain behavioral and pathological domains.

We next investigated whether intermittent reprogramming using YFs induces meaningful changes in one of the brain’s core functions: synaptic plasticity and connectivity. To our knowledge, this is the first demonstration that tightly controlled, intermittent YF expression enhances neuronal firing and synchrony in hippocampal neural networks. A classical explanation is that YF expression promotes rejuvenation of the targeted neuronal tissue, as previously suggested [23, 26, 28, 36]. However, to better understand the underlying mechanisms, we performed in vivo mass spectrometry analysis. This revealed that multiple synaptic proteins such as GluA1, GluA3, SAP102, GluN1, GluN2A, GluN2B, PYK2, and CaMKIIα were dysregulated in P301S mice. Notably, reprogramming restored the expression of an entire functional unit: the NMDAR macro-complex, including all NMDAR subunits and PYK2. NMDARs are key therapeutic targets in Alzheimer’s disease (AD) [10] and play a central role in synaptic function and plasticity. In this context, we found that the major downstream effector of NMDAR signaling, PYK2, was downregulated in P301S mice and restored following reprogramming. PYK2 is a non-receptor tyrosine kinase implicated in synaptic plasticity, memory formation, and emotional regulation [13]. Importantly, PYK2 has emerged as a promising molecule in AD research, with several studies identifying single-nucleotide polymorphisms (SNPs) in the *PTK2B* gene as genetic risk factors for AD [16, 49]. Furthermore, PYK2 has been shown to modulate Tau pathology [50], potentially through its regulation of GSK3β activity [44, 51], a key kinase responsible for the hyperphosphorylation and pathological accumulation of Tau [45]. Additionally, NMDAR macro-complexes have been shown to regulate hippocampal neural synchrony [52, 53], which aligns with our in vitro and in vivo findings following intermittent reprogramming. Although our study did not explore in depth the proposed NMDAR macro-complexes (NMDAR–PYK2–GSK3β-Tau), previous work from our group and others has demonstrated that manipulating these molecules individually is sufficient to exert beneficial effects in AD mouse models. Specifically, we have shown that overexpressing PYK2 in hippocampal neurons of the 5xFAD mouse model improves cognitive performance [54]. Moreover, pharmacological inhibition of GSK3β with meridianins (a highly specific marine compound) ameliorates neuroinflammation and enhances declarative memory in the same model [55]. Overall, current support for our NMDAR macro-complexes hypothesis is mainly correlative and requires further experimental validation.

All aforementioned molecular changes may be partially driven by epigenetic remodeling as suggested by our analysis of histone modifications. Specifically, we observed normalization of aberrant methylation and acetylation of histone H3 at lysine 9 (H3K9), which are known to be altered in AD [39, 56]. Changes in H3K9ac may represent an important mechanism through which the partial and intermittent expression of Yamanaka factors (YFs) in principal hippocampal neurons drives the observed improvements. Tau pathology is known to restructure the H3K9ac landscape in the aging brain in ways linked to alterations in 3D chromatin organization, accompanied by concordant transcriptional changes [57]. Consistent with this, Tau‑dependent changes in H3K9ac can enhance CDK5 expression and thereby promote Tau hyperphosphorylation [58]. Intermittent expression of YFs may modulate and correct such epigenetic disruptions. For example, SOX2 (a YF) regulates H3K9ac, generally acting to promote this active chromatin mark and maintain chromatin in an open and accessible state [59]. Similarly, KLF4 (another YF) has been shown to function in coordination with p300 as a histone acetyltransferase (HAT) modulator, targeting H3K9 to facilitate transcriptional activation [60]. OCT4 (the third YF) also contributes to H3K9 acetylation [61]. Importantly, H3K9 acetylation levels correlate with proper synaptic plasticity and memory performance in the contextual fear conditioning paradigm [62]. Thus, YF‑dependent restoration of H3K9 acetylation levels in i4F‑P301S‑Dox mice, compared with i4F‑P301S‑Veh mice, could underlie the observed functional improvements.

Although neural synchrony was rescued in i4F‑P301S + Dox mice, LTP remained equally impaired in both i4F‑P301S + Veh and i4F‑P301S + Dox groups. In this regard, although LTP has traditionally been considered a gold‑standard correlate of memory performance in mice [63], we and others have demonstrated that cognitive improvement does not always coincide with enhanced or rescued LTP [42, 64]. In addition, key methodological variables must be considered, such as the LTP induction protocol used (high‑frequency stimulation vs. theta‑burst stimulation) and the specific hippocampal pathway targeted (CA3–CA1 vs. DG–CA3). Altogether, these considerations support the view that LTP is one (though not the only) mechanism underlying synaptic plasticity and memory storage, as highlighted in seminal reviews [64]. Thus, we consider that the improved memory performance observed in our study correlates more robustly with the restoration of neural synchrony in hippocampal circuits, as previously shown [65]. In conclusion, the LTP discrepancy remains to be elucidated to confirm this hypothesis.

Previously, we observed that in vivo intermittent reprogramming of principal hippocampal neurons could modulate the activity of microglia surrounding amyloid plaques in the 5xFAD mouse model of AD [25]. In the current study, although our mass spectrometry analysis did not reveal major changes in immune- or neuroinflammation-related pathways, we nonetheless observed a marked restoration of microglial (but not astroglial) morphological parameters. This may reflect a non-cell-autonomous, indirect effect driven by general neuronal improvements, as previously reported in other AD models [66]. In the context of AD, improved microglial morphology may indicate a shift from a disease-associated microglial (DAM) phenotype toward a more homeostatic state, potentially reducing aberrant synapse engulfment [67]. This shift could contribute to the observed rescue of spine density loss in reprogrammed P301S mice.

Finally, our study has several limitations that limit mechanistic interpretation and implies that some of our explanations should be interpreted with caution. We observed only subtle behavioral improvements in female P301S mice. Given the notable molecular and neuropathological improvements observed in females, it is plausible that the behavioral paradigms we used were not optimal for detecting significant changes in this sex. Also, in our study we treated mice chronically with doxycycline for months, although such reports employed therapeutic doses 100–200 times more concentrated than ours [68, 69], we cannot completely rule out a potential effect by doxycycline alone even though we used all the appropriate controls. Additionally, certain sources of variability were not controlled, such as the individual drink intake for each mouse. Finally, although our findings are promising, they should be interpreted with caution. Future research should investigate in vivo intermittent partial reprogramming in human‑based systems such as organoids or human pluripotent stem cell–derived neurons and employ complementary technologies to better evaluate safety, including long‑term effects, and potential suitability for clinical translation. Also, future research should address whether reprogramming and rejuvenating cortical neurons, or co‑reprogramming principal neuronal populations across more than one brain region (for example, hippocampal and cortical neurons), could exert similar or even greater phenotypic rescue effects than those observed in the present study.

## Materials and methods

### Animals

For in vivo experiments in adult mice, we used the previously reported P301S transgenic line [70], which was crossed with the i4F‑A (i4F) mouse line [25] to generate the i4F‑P301S model. Homozygous i4F‑A mice were specifically bred with heterozygous P301S animals. This crossing strategy produced offspring that were all heterozygous for i4F‑A, while 50% also carried the P301S transgene in a heterozygous state. These animals were subsequently used for experimental studies. In addition, we analyzed the parental lines (i4F‑A and P301S) together with the newly generated double‑transgenic line (i4F‑P301S) for viability, detecting no alterations in fertility, embryonic development, or perinatal/early postnatal survival (S1). Mice were housed with ad libitum access to food and water in a controlled environment (19–22°C, 40%–60% humidity) under a 12 h light/dark cycle. In all experiments, doxycycline (Dox; BioChemica) was provided in the drinking water supplemented with 7.5% sucrose as previously described [25]. All procedures were conducted in accordance with ethical guidelines (Declaration of Helsinki, NIH Publication no. 85–23 revised 1985, European Community guidelines) and approved by the University of Barcelona ethics committee (CEEA: 55/21) and the regional authorities (Generalitat de Catalunya: 11,559).

### Acute brain slice preparation and patch-clamp recordings

Patch‑clamp recordings were conducted as previously reported [71]. Brain slices were visualized using a fluorescence microscope equipped with IR‑DIC optics (Olympus BX51). The holding potential was maintained at −70 mV for EPSCs and at 0 mV for IPSCs. Signals were recorded with a MultiClamp 700B amplifier coupled to a Digidata 1550 digitizer (Molecular Devices), acquired using Clampex 10.3 software, and analyzed with Clampfit (Molecular Devices, Sunnyvale, CA). Data were sampled at 10 kHz and low‑pass filtered at 2 kHz.

### 3D primary neuronal cultures

Modular neuronal networks (MoNNets) in three‑dimensional neurospheres were generated as described elsewhere [30]. We used the inducible four‑factor (i4F‑Rosa) mouse line, which enables YF activation through a Tet‑ON system [25]. From binarized signals, the following parameters were extracted: mean activity rate, peak duration, and average pairwise Pearson correlation between neurospheres. Time‑lapse recordings were performed at DIV19, DIV24, and DIV26.

### Adeno-associated viral vectors

We generated a viral construct to restrict rtTA expression to principal neurons using the AAV8 capsid and the Synapsin I promoter, enabling OSKM induction via a Tet‑ON system. The vector pAAV8[TetOn]-TRE > ZsGreen1-rev(SYN1 > tTS:T2A:rtTA) was injected into the hippocampus of adult i4F (control) and i4F‑P301S male and female mice at 8 weeks of age (Fig. 2a). At 12 weeks, mice received either vehicle (Veh; 7.5% sucrose) or doxycycline (Dox; 0.2 mg/mL in 7.5% sucrose) in drinking water, administered 3 days per week for 6 months. During the final month, at 8 months of age, animals underwent a comprehensive behavioral test battery. At the end of treatment, mice were sacrificed for subsequent biochemical and histological analyses.

### Stereotaxic surgeries

During isoflurane anesthesia (2% induction, 1.5% maintenance; 2% oxygen), bilateral hippocampal injections of pAAV8[TetOn]-TRE > ZsGreen1-rev(SYN1 > tTS:T2A:rtTA (2 × 10^13^ GC/ml in PBS; Vector Builder, ID: VB210109-1009yyx) were performed as previously described [25]. Following surgery, animals were returned to their home cages for 4 weeks prior to initiating doxycycline (Dox) treatment. Dox administration (0.2 mg/kg, 4 days/week) began at 12 weeks of age and continued until 36 weeks.

### Behavioral assessment

Behavioral assessments, including the open field, Y‑maze alternation, and forced swim tests, were conducted as described elsewhere [25]. *Contextual Fear Conditioning*: A black chamber (25 × 25 × 25 cm; Panlab, Spain) was used. On Day 1, mice were allowed to habituate for 5 min, followed by a 30 s tone (90 dB). During the last 2 s of the tone, a 0.4 mA foot shock was delivered (1–2 s), after which animals remained in the chamber for an additional 30 s. This sequence was repeated twice, and mice stayed in the chamber for a further 2 min before removal. On Day 2, contextual memory was evaluated by placing mice back into the chamber for 5 min without tone presentation, and freezing behavior was recorded. Animal tracking and data acquisition were performed using Packwin V2.0.08 software (Panlab, Spain).

### Immunofluorescence and microscope imaging

Mice were euthanized by cervical dislocation. Brain processing and immunofluorescence were carried out as previously described [55]. Brain sections were incubated overnight at 4 °C under gentle agitation with the following primary antibodies in blocking buffer: anti‑Phospho‑Tau (Ser202/Thr205; mouse, 1:300, #MN1020, Invitrogen), anti‑GFAP (rabbit, 1:300, #z0334, DAKO), anti‑IBA1 (goat, 1:300, #Ab5076, Abcam), anti‑H3K9me3 (rabbit, 1:300, #Ab8898, Abcam), anti‑H3K9Ac (rabbit, 1:300, #9649S, Cell Signaling), anti‑NeuN (mouse, 1:300, MAB377, Millipore/Chemicon), anti‑OLIG2 (rabbit, 1:500, AB9610, Millipore), anti‑PYK2 (rabbit, 1:500, P3902, Sigma), anti‑CD68 (rabbit, 1:300, ab125212, Abcam), anti‑TNFα (mouse, 1:200, ab1793, Abcam), anti‑NANOG (rabbit, 1:300, NB100‑58,842, Novus), and anti‑Klf4 (goat, 1:300, #AF3158, R&D Systems). Imaging was performed using a Leica TCS SP5 confocal microscope with a 40 × objective (NA) and a 2‑Airy disk pinhole. Z‑stacks (1024 × 1024 pixels) were acquired across the full volume. Image analysis was conducted in ImageJ (v1.53f), using specific macros to quantify IBA1‑ and GFAP‑positive cells [72] while mean fluorescence intensity was measured for the remaining markers [25] as described elsewhere.

### Mass spectrometry

Protein extraction, quantitation, digestion, peptide purification and LC–MS analysis was performed as previously described [73]. The Perseus software (version 1.6.15.0) was used for statistical analysis and data visualization. A filter of 70% valid values at least in one group was applied and data were imputated based on the normal distribution and normalized using the “Width adjustment” method. Statistical analysis was performed using “Two-sample tests” for the T-test of two selected experimental groups, based on permutation-based FDR statistics (250 permutations; FDR 1⁄4 0.07; S0 1⁄4 0.1). The resulting matrixes were exported containing the p-value (-Log) and fold-change (Log2) columns that, after its transformation into linear scale, were filtered by *p* < 0.05 and 30% of significance and fold-change respectively.

### Western blotting

Mice (n = 3–4 per group) were euthanized by cervical dislocation. Immunoblotting was carried out as described elsewhere [55]. Membranes were incubated overnight at 4 °C with anti‑GSK3β (27C10; rabbit, 1:1000, #9315S, Cell Signaling) and anti‑phospho‑GSK3β (Ser9; rabbit, 1:1000, #9336S, Cell Signaling). A mouse monoclonal anti‑TubA4A (TubA1; 1:50,000, Sigma‑Aldrich, #T‑9026) served as loading control. Protein bands were detected using a ChemiDoc™ Imaging System (Bio‑Rad).

### Golgi-cox staining

Fresh left brain hemispheres were processed using the Golgi‑Cox method as previously described [42]. Spine density (spines/µm) was quantified using FIJI software (Wayne Rasband, NIH, RRID:SCR_003070). A minimum of 4–7 mice per group were analyzed, with 45–50 dendrites evaluated per animal.

### Electrophysiological studies

Horizontal mouse brain slices (350 µm) were cut on a vibratome (Microm HM 650 V, Thermo Scientific) in ice‑cold, carbogenated aCSF (95% O₂/5% CO₂). Slices recovered for 15 min at 34 °C in oxygenated recovery solution [74], and were then maintained in oxygenated aCSF at room temperature for ≥ 1 h. For MEA recordings, slices were transferred to MEA dishes, fully submerged in oxygenated aCSF at 37 °C, and signals were acquired with a 60‑channel USB‑MEA60‑inv system (Multi Channel Systems). We used 60MEA200/30iR‑ITO arrays (8 × 8 layout; 60 planar electrodes) positioned over the hippocampal formation.

Pontaneous activity was recorded for 5 min from 58 electrodes in CA1 (10 kHz sampling). Spikes were defined as negative events exceeding −20 µV, as in [75]. Bursts were quantified with the MaxInterval method [76] (max start/end ISI 50 ms; min inter‑burst interval 20 ms; min burst duration 20 ms; ≥ 5 spikes/burst). Field postsynaptic currents (fPSCs) were recorded in CA1 after stimulation of CA3 afferents (4.36 mm interaural, 1.7 mm lateral, 2 mm posterior to bregma; Fig. 6b). fPSCs were evoked with single monopolar biphasic pulses; input/output curves were built using trains of three identical positive–negative pulses at increasing intensities (500–4000 mV) [74]. Long-term potentiation (LTP) was induced by HFS (one 1‑s train at 100 Hz). fPSC amplitudes were measured throughout. Acquisition and processing were performed in MC Rack (Multi Channel Systems), and slice placement on the array was confirmed with a digital camera.

### Statistics

Statistical analyses were performed using GraphPad Prism v.10 (GraphPad Software, La Jolla, CA, USA). Comparisons between two groups were assessed using a two‑tailed unpaired Student’s t‑test, whereas multiple group analyses were conducted using one‑way ANOVA with Tukey’s post hoc test or two‑way ANOVA with Bonferroni correction, as appropriate. Normality was evaluated using the D’Agostino–Pearson omnibus test; when data were not normally distributed, Mann–Whitney or Dunn’s tests were applied. Data are presented as mean ± standard error of the mean (SEM). Sample sizes were determined by power analysis (α = 0.05, estimated σ = 1, power = 75%). All mice generated were included in pre‑planned experiments and randomly assigned to experimental groups. Data collection and analysis were conducted in a blinded/randomized manner. Littermates served as controls, with 3–5 independent litters analyzed per experiment. Statistical significance was set at *p* < 0.05.

## Supplementary Information

Supplementary Material 1.

Supplementary Material 2.

## Data Availability

MS data and search results files were deposited in the Proteome Xchange Consortium via the JPOST partner repository (https://repository.jpostdb.org) [77] with the identifier PXD066415 for ProteomeXchange and JPST003956 for jPOST (for reviewers https://repository.jpostdb.org/preview/1201414225687f8f1c76edd; Access key: 9229). This paper does not report original code. Any additional information required to reanalyze the data reported in this paper is available from the lead contact upon request. Rest of data and materials will be made available upon reasonable request.

## References

[1] Fox J, Mearns ES, Li J, Rosettie KL, Majda T, Lin H, et al. Indirect costs of Alzheimer’s disease: unpaid caregiver burden and patient productivity loss. Value Health. 2025;28:519–26. 10.1016/j.jval.2024.10.3851. 39571734 10.1016/j.jval.2024.10.3851

[2] D’Onofrio G, Sancarlo D, Panza F, Copetti M, Cascavilla L, Paris F, et al. Neuropsychiatric Symptoms and Functional Status in Alzheimer’s Disease and Vascular Dementia Patients. Curr Alzheimer Res. 2012;9:759–71. 10.2174/156720512801322582. 22715983 10.2174/156720512801322582

[3] DeTure MA, Dickson DW. The neuropathological diagnosis of Alzheimer’s disease. Mol Neurodegener. 2019;14:32. 10.1186/s13024-019-0333-5. 31375134 10.1186/s13024-019-0333-5 PMC6679484

[4] Graff-Radford J, Yong KXX, Apostolova LG, Bouwman FH, Carrillo M, Dickerson BC, et al. New insights into atypical Alzheimer’s disease in the era of biomarkers. Lancet Neurol. 2021;20:222–34. 10.1016/S1474-4422(20)30440-3. 33609479 10.1016/S1474-4422(20)30440-3 PMC8056394

[5] Zhang J, Zhang Y, Wang J, Xia Y, Zhang J, Chen L. Recent advances in Alzheimer’s disease: mechanisms, clinical trials and new drug development strategies. Signal Transduct Target Ther. 2024;9:211. 10.1038/s41392-024-01911-3. 39174535 10.1038/s41392-024-01911-3 PMC11344989

[6] Passamonti L, Tsvetanov KA, Jones PS, Bevan-Jones WR, Arnold R, Borchert RJ, et al. Neuroinflammation and functional connectivity in Alzheimer’s disease: interactive influences on cognitive performance. J Neurosci. 2019;39:7218–26. 10.1523/JNEUROSCI.2574-18.2019. 31320450 10.1523/JNEUROSCI.2574-18.2019 PMC6733539

[7] Skaper SD, Facci L, Zusso M, Giusti P. Synaptic plasticity, dementia and Alzheimer disease. CNS Neurol Disord Drug Targets. 2017;16:220–33. 10.2174/1871527316666170113120853. 28088900 10.2174/1871527316666170113120853

[8] Ranasinghe KG, Kudo K, Hinkley L, Beagle A, Lerner H, Mizuiri D, et al. Neuronal synchrony abnormalities associated with subclinical epileptiform activity in early-onset Alzheimer’s disease. Brain. 2022;145:744–53. 10.1093/brain/awab442. 34919638 10.1093/brain/awab442 PMC9630715

[9] Ranasinghe KG, Cha J, Iaccarino L, Hinkley LB, Beagle AJ, Pham J, et al. Neurophysiological signatures in Alzheimer’s disease are distinctly associated with TAU, amyloid-β accumulation, and cognitive decline. Sci Transl Med. 2020;12. 10.1126/scitranslmed.aaz4069. 10.1126/scitranslmed.aaz4069 PMC7138514 32161102

[10] Liu J, Chang L, Song Y, Li H, Wu Y. The Role of NMDA Receptors in Alzheimer’s Disease. Front Neurosci. 2019;13. 10.3389/fnins.2019.00043. 10.3389/fnins.2019.00043 PMC6375899 30800052

[11] Wang H, Yang J, Schneider JA, De Jager PL, Bennett DA, Zhang H-Y. Genome-wide interaction analysis of pathological hallmarks in Alzheimer’s disease. Neurobiol Aging. 2020;93:61–8. 10.1016/j.neurobiolaging.2020.04.025. 32450446 10.1016/j.neurobiolaging.2020.04.025 PMC9795865

[12] Chung J, Wang X, Maruyama T, Ma Y, Zhang X, Mez J, et al. Genome‐wide association study of Alzheimer’s disease endophenotypes at prediagnosis stages. Alzheimers Dement. 2018;14:623–33. 10.1016/j.jalz.2017.11.006. 29274321 10.1016/j.jalz.2017.11.006 PMC5938137

[13] de Pins B, Mendes T, Giralt A, Girault J-A. The Non-receptor Tyrosine Kinase Pyk2 in Brain Function and Neurological and Psychiatric Diseases. Front Synaptic Neurosci. 2021;13. 10.3389/fnsyn.2021.749001. 10.3389/fnsyn.2021.749001 PMC8527176 34690733

[14] Bradley CA, Peineau S, Taghibiglou C, Nicolas CS, Whitcomb DJ, Bortolotto ZA, et al. A pivotal role of GSK-3 in synaptic plasticity. Front Mol Neurosci. 2012;5. 10.3389/fnmol.2012.00013. 10.3389/fnmol.2012.00013 PMC3279748 22363262

[15] Hernandez F, Lucas JJ, Avila J. GSK3 and Tau: two convergence points in Alzheimer’s Disease. J Alzheimers Dis. 2012;33:S141–4. 10.3233/JAD-2012-129025. 10.3233/JAD-2012-129025 22710914

[16] Lambert J-C, Ibrahim-Verbaas CA, Harold D, Naj AC, Sims R, Bellenguez C, et al. Meta-analysis of 74,046 individuals identifies 11 new susceptibility loci for Alzheimer’s disease. Nat Genet. 2013;45:1452–8. 10.1038/ng.2802. 24162737 10.1038/ng.2802 PMC3896259

[17] Brody AH, Nies SH, Guan F, Smith LM, Mukherjee B, Salazar SA, et al. Alzheimer risk gene product Pyk2 suppresses tau phosphorylation and phenotypic effects of tauopathy. Mol Neurodegener. 2022;17:32. 10.1186/s13024-022-00526-y. 35501917 10.1186/s13024-022-00526-y PMC9063299

[18] Wagatsuma N, von der Heydt R, Niebur E. Spike synchrony generated by modulatory common input through NMDA-type synapses. J Neurophysiol. 2016;116:1418–33. 10.1152/jn.01142.2015. 27486111 10.1152/jn.01142.2015 PMC5040377

[19] Liu Y, Tan Y, Zhang Z, Yi M, Zhu L, Peng W. The interaction between ageing and Alzheimer’s disease: insights from the hallmarks of ageing. Transl Neurodegener. 2024;13:7. 10.1186/s40035-024-00397-x. 38254235 10.1186/s40035-024-00397-x PMC10804662

[20] Hou Y, Dan X, Babbar M, Wei Y, Hasselbalch SG, Croteau DL, et al. Ageing as a risk factor for neurodegenerative disease. Nat Rev Neurol. 2019;15:565–81. 10.1038/s41582-019-0244-7. 31501588 10.1038/s41582-019-0244-7

[21] Leparulo A, Bisio M, Redolfi N, Pozzan T, Vassanelli S, Fasolato C. Accelerated aging characterizes the early stage of Alzheimer’s Disease. Cells. 2022;11:238. 10.3390/cells11020238. 35053352 10.3390/cells11020238 PMC8774248

[22] Cipriano A, Moqri M, Maybury-Lewis SY, Rogers-Hammond R, de Jong TA, Parker A, et al. Mechanisms, pathways and strategies for rejuvenation through epigenetic reprogramming. Nat Aging. 2023;4:14–26. 10.1038/s43587-023-00539-2. 38102454 10.1038/s43587-023-00539-2 PMC11058000

[23] Simpson DJ, Olova NN, Chandra T. Cellular reprogramming and epigenetic rejuvenation. Clin Epigenetics. 2021;13:170. 10.1186/s13148-021-01158-7. 34488874 10.1186/s13148-021-01158-7 PMC8419998

[24] Wei Z, Yang Y, Zhang P, Andrianakos R, Hasegawa K, Lyu J, et al. Klf4 interacts directly with Oct4 and Sox2 to promote reprogramming. Stem Cells. 2009;27:2969–78. 10.1002/stem.231. 19816951 10.1002/stem.231

[25] Shen Y-R, Zaballa S, Bech X, Sancho-Balsells A, Rodríguez-Navarro I, Cifuentes-Díaz C, et al. Expansion of the neocortex and protection from neurodegeneration by in vivo transient reprogramming. Cell Stem Cell. 2024;31:1741-1759.e8. 10.1016/j.stem.2024.09.013. 39426381 10.1016/j.stem.2024.09.013

[26] Neumann B, Segel M, Ghosh T, Zhao C, Tourlomousis P, Young A, et al. Myc determines the functional age state of oligodendrocyte progenitor cells. Nat Aging. 2021;1:826–37. 10.1038/s43587-021-00109-4. 37117631 10.1038/s43587-021-00109-4

[27] Antón-Fernández A, Roldán-Lázaro M, Vallés-Saiz L, Ávila J, Hernández F. In vivo cyclic overexpression of Yamanaka factors restricted to neurons reverses age-associated phenotypes and enhances memory performance. Commun Biol. 2024;7:631. 10.1038/s42003-024-06328-w. 38789561 10.1038/s42003-024-06328-w PMC11126596

[28] Xu L, Ramirez-Matias J, Hauptschein M, Sun ED, Lunger JC, Buckley MT, et al. Restoration of neuronal progenitors by partial reprogramming in the aged neurogenic niche. Nat Aging. 2024;4:546–67. 10.1038/s43587-024-00594-3. 38553564 10.1038/s43587-024-00594-3 PMC12036604

[29] Drake SS, Mohammadnia A, Zaman A, Gianfelice C, Heale K, Groh AMR, et al. Cellular rejuvenation protects neurons from inflammation-mediated cell death. Cell Rep. 2025;44:115298. 10.1016/j.celrep.2025.115298. 39937646 10.1016/j.celrep.2025.115298

[30] Ballasch I, López-Molina L, Galán-Ganga M, Sancho-Balsells A, Rodríguez-Navarro I, Borràs-Pernas S, et al. Alterations of the IKZF1-IKZF2 tandem in immune cells of schizophrenia patients regulate associated phenotypes. J Neuroinflammation. 2024;21:326. 10.1186/s12974-024-03320-3. 39695786 10.1186/s12974-024-03320-3 PMC11658472

[31] Meftah S, Gan J. Alzheimer’s disease as a synaptopathy: Evidence for dysfunction of synapses during disease progression. Front Synaptic Neurosci. 2023;15. 10.3389/fnsyn.2023.1129036. 10.3389/fnsyn.2023.1129036 PMC10033629 36970154

[32] Koivisto H, Ytebrouck E, Carmans S, Naderi R, Miettinen PO, Roucourt B, et al. Progressive age-dependent motor impairment in human tau P301S overexpressing mice. Behav Brain Res. 2019;376:112158. 10.1016/j.bbr.2019.112158. 31442549 10.1016/j.bbr.2019.112158

[33] Takeuchi H, Iba M, Inoue H, Higuchi M, Takao K, Tsukita K, et al. P301S Mutant Human Tau Transgenic Mice Manifest Early Symptoms of Human Tauopathies with Dementia and Altered Sensorimotor Gating. PLoS ONE. 2011;6:e21050. 10.1371/journal.pone.0021050. 21698260 10.1371/journal.pone.0021050 PMC3115982

[34] Suemaru S, Hashimoto K, Suemaru K, Maeba Y, Matsushita N, Ota Z. Hyperkinesia, plasma corticotropin releasing hormone and ACTH in senile dementia. NeuroReport. 1991;2:337–40. 10.1097/00001756-199106000-00009. 1655106 10.1097/00001756-199106000-00009

[35] Goedert M, Jakes R, Vanmechelen E. Monoclonal antibody AT8 recognises tau protein phosphorylated at both serine 202 and threonine 205. Neurosci Lett. 1995;189:167–70. 10.1016/0304-3940(95)11484-E. 7624036 10.1016/0304-3940(95)11484-e

[36] Horvath S, Lacunza E, Mallat MC, Portiansky EL, Gallardo MD, Brooke RT, et al. Cognitive rejuvenation in old rats by hippocampal OSKM gene therapy. GeroScience. 2024;47:809–23. 10.1007/s11357-024-01269-y. 39037528 10.1007/s11357-024-01269-y PMC11872836

[37] Wang W, Cao Q, Tan T, Yang F, Williams JB, Yan Z. Epigenetic treatment of behavioral and physiological deficits in a tauopathy mouse model. Aging Cell. 2021;20. 10.1111/acel.13456. 10.1111/acel.13456 PMC8520711 34547169

[38] Kim J, Selvaraji S, Kang SW, Lee WT, Chen CL-H, Choi H, et al. Cerebral transcriptome analysis reveals age-dependent progression of neuroinflammation in P301S mutant tau transgenic male mice. Brain Behav Immun. 2019;80:344–57. 10.1016/j.bbi.2019.04.011. 30980950 10.1016/j.bbi.2019.04.011

[39] Williams JB, Cao Q, Wang W, Lee Y-H, Qin L, Zhong P, et al. Inhibition of histone methyltransferase Smyd3 rescues NMDAR and cognitive deficits in a tauopathy mouse model. Nat Commun. 2023;14:91. 10.1038/s41467-022-35749-6. 36609445 10.1038/s41467-022-35749-6 PMC9822922

[40] Guan Y, Wang R, Li X, Zou H, Yu W, Liang Z, et al. Astrocytes constitute the major TNF-α-producing cell population in the infarct cortex in dMCAO rats receiving intravenous MSC infusion. Biomed Pharmacother. 2021;142:111971. 10.1016/j.biopha.2021.111971. 34343893 10.1016/j.biopha.2021.111971

[41] Chistiakov DA, Killingsworth MC, Myasoedova VA, Orekhov AN, Bobryshev YV. CD68/macrosialin: not just a histochemical marker. Lab Invest. 2017;97:4–13. 10.1038/labinvest.2016.116. 27869795 10.1038/labinvest.2016.116

[42] Giralt A, Brito V, Chevy Q, Simonnet C, Otsu Y, Cifuentes-Díaz C, et al. Pyk2 modulates hippocampal excitatory synapses and contributes to cognitive deficits in a Huntington’s disease model. Nat Commun. 2017;8:15592. 10.1038/ncomms15592. 28555636 10.1038/ncomms15592 PMC5459995

[43] Sayas CL, Ariaens A, Ponsioen B, Moolenaar WH. GSK-3 is activated by the tyrosine kinase Pyk2 during LPA 1 -mediated neurite retraction. Mol Biol Cell. 2006;17:1834–44. 10.1091/mbc.e05-07-0688. 16452634 10.1091/mbc.E05-07-0688 PMC1415316

[44] Hartigan JA, Xiong W-C, Johnson GVW. Glycogen synthase kinase 3β is tyrosine phosphorylated by PYK2. Biochem Biophys Res Commun. 2001;284:485–9. 10.1006/bbrc.2001.4986. 11394906 10.1006/bbrc.2001.4986

[45] Chakraborty P, Ibáñez de Opakua A, Purslow JA, Fromm SA, Chatterjee D, Zachrdla M, et al. GSK3β phosphorylation catalyzes the aggregation of tau into Alzheimer’s disease-like filaments. Proc Natl Acad Sci. 2024;121. 10.1073/pnas.2414176121. 10.1073/pnas.2414176121 PMC11670061 39693350

[46] Yoshiyama Y, Kojima A, Itoh K, Uchiyama T, Arai K. Anticholinergics boost the pathological process of neurodegeneration with increased inflammation in a tauopathy mouse model. Neurobiol Dis. 2012;45:329–36. 10.1016/j.nbd.2011.08.017. 21889983 10.1016/j.nbd.2011.08.017

[47] Sun Y, Guo Y, Feng X, Jia M, Ai N, Dong Y, et al. The behavioural and neuropathologic sexual dimorphism and absence of MIP-3α in tau P301S mouse model of Alzheimer’s disease. J Neuroinflammation. 2020;17:72. 10.1186/s12974-020-01749-w. 32093751 10.1186/s12974-020-01749-w PMC7041244

[48] Goodwin LO, Splinter E, Davis TL, Urban R, He H, Braun RE, et al. Large-scale discovery of mouse transgenic integration sites reveals frequent structural variation and insertional mutagenesis. Genome Res. 2019;29:494–505. 10.1101/gr.233866.117. 30659012 10.1101/gr.233866.117 PMC6396414

[49] Li Y-Q, Tan M-S, Wang H-F, Tan C-C, Zhang W, Zheng Z-J, et al. Common variant in PTK2B is associated with late-onset Alzheimer’s disease: a replication study and meta-analyses. Neurosci Lett. 2016;621:83–7. 10.1016/j.neulet.2016.04.020. 27080426 10.1016/j.neulet.2016.04.020

[50] Dourlen P, Fernandez-Gomez FJ, Dupont C, Grenier-Boley B, Bellenguez C, Obriot H, et al. Functional screening of Alzheimer risk loci identifies PTK2B as an in vivo modulator and early marker of Tau pathology. Mol Psychiatry. 2017;22:874–83. 10.1038/mp.2016.59. 27113998 10.1038/mp.2016.59 PMC5444024

[51] Gao C, Chen G, Kuan S-F, Zhang DH, Schlaepfer DD, Hu J. FAK/PYK2 promotes the Wnt/β-catenin pathway and intestinal tumorigenesis by phosphorylating GSK3β. Elife. 2015;4. 10.7554/eLife.10072. 10.7554/eLife.10072 PMC4558782 26274564

[52] Fellin T, Pascual O, Gobbo S, Pozzan T, Haydon PG, Carmignoto G. Neuronal synchrony mediated by astrocytic glutamate through activation of extrasynaptic NMDA receptors. Neuron. 2004;43:729–43. 10.1016/j.neuron.2004.08.011. 15339653 10.1016/j.neuron.2004.08.011

[53] Ruggiero A, Heim LR, Susman L, Hreaky D, Shapira I, Katsenelson M, et al. NMDA receptors regulate the firing rate set point of hippocampal circuits without altering single-cell dynamics. Neuron. 2025;113:244-259.e7. 10.1016/j.neuron.2024.10.014. 39515323 10.1016/j.neuron.2024.10.014

[54] Giralt A, de Pins B, Cifuentes-Díaz C, López-Molina L, Farah AT, Tible M, et al. PTK2B/Pyk2 overexpression improves a mouse model of Alzheimer’s disease. Exp Neurol. 2018;307:62–73. 10.1016/j.expneurol.2018.05.020. 29803828 10.1016/j.expneurol.2018.05.020

[55] Rodríguez-Urgellés E, Sancho-Balsells A, Chen W, López-Molina L, Ballasch I, Castillo I del, et al. Meridianins Rescue Cognitive Deficits, Spine Density and Neuroinflammation in the 5xFAD Model of Alzheimer’s Disease. Front Pharmacol. 2022;13. 10.3389/fphar.2022.791666. 10.3389/fphar.2022.791666 PMC8908099 35281935

[56] Jiang Y, Jakovcevski M, Bharadwaj R, Connor C, Schroeder FA, Lin CL, et al. Setdb1 histone methyltransferase regulates mood-related behaviors and expression of the NMDA receptor subunit NR2B. J Neurosci. 2010;30:7152–67. 10.1523/JNEUROSCI.1314-10.2010. 20505083 10.1523/JNEUROSCI.1314-10.2010 PMC2893142

[57] Klein H-U, McCabe C, Gjoneska E, Sullivan SE, Kaskow BJ, Tang A, et al. Epigenome-wide study uncovers large-scale changes in histone acetylation driven by Tau pathology in aging and Alzheimer’s human brains. Nat Neurosci. 2019;22:37–46. 10.1038/s41593-018-0291-1. 30559478 10.1038/s41593-018-0291-1 PMC6516529

[58] Yu C-C, Jiang T, Yang A-F, Du Y-J, Wu M, Kong L-H. Epigenetic modulation on Tau phosphorylation in Alzheimer’s disease. Neural Plast. 2019;2019:1–12. 10.1155/2019/6856327. 10.1155/2019/6856327 PMC6481020 31093272

[59] Amador-Arjona A, Cimadamore F, Huang C-T, Wright R, Lewis S, Gage FH, et al. SOX2 primes the epigenetic landscape in neural precursors enabling proper gene activation during hippocampal neurogenesis. Proc Natl Acad Sci U S A. 2015;112:E1936–45. 10.1073/pnas.1421480112. 25825708 10.1073/pnas.1421480112 PMC4403144

[60] Evans PM, Zhang W, Chen X, Yang J, Bhakat KK, Liu C. Krüppel-like Factor 4 is acetylated by p300 and regulates gene transcription via modulation of histone acetylation. J Biol Chem. 2007;282:33994–4002. 10.1074/jbc.M701847200. 17908689 10.1074/jbc.M701847200

[61] Wu Y, Manna AK, Li L, Shen Z, Handa H, Chandrasekharan MB, et al. Jade1 and the HBO1 histone acetyltransferase complex are spatial-selective cofactors of the pluripotency transcription factor Oct4. J Biol Chem. 2025;301:110859. 10.1016/j.jbc.2025.110859. 41489900 10.1016/j.jbc.2025.110859 PMC12664036

[62] Peixoto L, Abel T. The role of histone acetylation in memory formation and cognitive impairments. Neuropsychopharmacology. 2013;38:62–76. 10.1038/npp.2012.86. 22669172 10.1038/npp.2012.86 PMC3521994

[63] Bliss TVP, Collingridge GL. A synaptic model of memory: long-term potentiation in the hippocampus. Nature. 1993;361:31–9. 10.1038/361031a0. 8421494 10.1038/361031a0

[64] Dringenberg HC. The history of <scp>long‐term</scp> potentiation as a memory mechanism: controversies, confirmation, and some lessons to remember. Hippocampus. 2020;30:987–1012. 10.1002/hipo.23213. 32442358 10.1002/hipo.23213

[65] Chen E-L, Chen T-W, Schreiter ER, Lin B-J. Synchronous ensembles of hippocampal CA1 pyramidal neurons during novel exploration. Elife. 2025;13. 10.7554/eLife.96718. 10.7554/eLife.96718 PMC12517686 41081764

[66] Pérez-Sisqués L, Sancho-Balsells A, Solana-Balaguer J, Campoy-Campos G, Vives-Isern M, Soler-Palazón F, et al. RTP801/REDD1 contributes to neuroinflammation severity and memory impairments in Alzheimer’s disease. Cell Death Dis. 2021;12:616. 10.1038/s41419-021-03899-y. 34131105 10.1038/s41419-021-03899-y PMC8206344

[67] Valiukas Z, Tangalakis K, Apostolopoulos V, Feehan J. Microglial activation states and their implications for Alzheimer’s Disease. J Prev Alzheimers Dis. 2025;12:100013. 10.1016/j.tjpad.2024.100013. 39800461 10.1016/j.tjpad.2024.100013 PMC12184064

[68] Balducci C, Forloni G. Doxycycline for Alzheimer’s Disease: fighting β-Amyloid Oligomers and neuroinflammation. Front Pharmacol. 2019;10:738. 10.3389/fphar.2019.00738. 31333460 10.3389/fphar.2019.00738 PMC6616274

[69] Paldino E, Balducci C, La Vitola P, Artioli L, D’Angelo V, Giampà C, et al. Neuroprotective effects of doxycycline in the R6/2 mouse model of Huntington’s Disease. Mol Neurobiol. 2020;57:1889–903. 10.1007/s12035-019-01847-8. 31879858 10.1007/s12035-019-01847-8 PMC7118056

[70] Cao Q, Kumar M, Frazier A, Williams JB, Zhao S, Yan Z. Longitudinal characterization of behavioral, morphological and transcriptomic changes in a tauopathy mouse model. Aging (Albany NY). 2023;15:11697–719. 10.18632/aging.205057. 37925173 10.18632/aging.205057 PMC10683589

[71] Wang L, Simms J, Peters CJ, Tynan-La Fontaine M, Li K, Gill TM, et al. TMEM16B calcium-activated chloride channels regulate action potential firing in lateral septum and aggression in male mice. J Neurosci. 2019;39:7102–17. 10.1523/JNEUROSCI.3137-18.2019. 31320449 10.1523/JNEUROSCI.3137-18.2019 PMC6733546

[72] Sancho-Balsells A, Borràs-Pernas S, Flotta F, Chen W, del Toro D, Rodríguez MJ, et al. Brain–gut photobiomodulation restores cognitive alterations in chronically stressed mice through the regulation of Sirt1 and neuroinflammation. J Affect Disord. 2024;354:574–88. 10.1016/j.jad.2024.03.075. 38490587 10.1016/j.jad.2024.03.075

[73] Sitjà-Roqueta L, Ngum NM, Zherebtsov EA, Küçükerden M, Givehchi M, Bova V, et al. Photoactivated adenylyl cyclase in cortical astrocytes promotes synaptic potentiation and reveals alterations in Huntington’s disease. iScience. 2025;28:113640. 10.1016/j.isci.2025.113640. 41146711 10.1016/j.isci.2025.113640 PMC12554215

[74] García-García E, Ramón-Lainez A, Conde-Berriozabal S, del Toro D, Escaramis G, Giralt A, et al. VPS13A knockdown impairs corticostriatal synaptic plasticity and locomotor behavior in a new mouse model of chorea-acanthocytosis. Neurobiol Dis. 2023;187:106292. 10.1016/j.nbd.2023.106292. 37714309 10.1016/j.nbd.2023.106292

[75] Sancho-Balsells A, García-García E, Flotta F, Chen W, Alberch J, Rodríguez MJ, et al. Meridianins inhibit GSK3β in vivo and improve behavioral alterations induced by chronic stress. Mar Drugs. 2022;20:648. 10.3390/md20100648. 36286471 10.3390/md20100648 PMC9605278

[76] Legendy CR, Salcman M. Bursts and recurrences of bursts in the spike trains of spontaneously active striate cortex neurons. J Neurophysiol. 1985;53:926–39. 10.1152/jn.1985.53.4.926. 3998798 10.1152/jn.1985.53.4.926

[77] Okuda S, Watanabe Y, Moriya Y, Kawano S, Yamamoto T, Matsumoto M, et al. jPOSTrepo: an international standard data repository for proteomes. Nucleic Acids Res. 2017;45:D1107–11. 10.1093/nar/gkw1080. 27899654 10.1093/nar/gkw1080 PMC5210561

