# Supplementary material for: Targeted neuronal reprogramming rescues memory and neural synchrony in Alzheimer’s disease
Source: Mol Biomed. 2026 Jun 10;7:87. doi: 10.1186/s43556-026-00481-w (PMC13253944; doi:10.1186/s43556-026-00481-w)
Supplement: Supplementary file 1 — Supplementary Material 1. [file 43556_2026_481_MOESM1_ESM.docx]

**Supplementary Figures**

**Targeted neuronal reprogramming rescues memory and neural synchrony in Alzheimer’s disease**

Marcos Galán-Ganga^1,2,3, †^, Irene Rodríguez-Navarro^1,2,3,†^, Sofía Zaballa^1,2,3, †^, Alba Ramón-Lainez^1,2,3^, Nerea Gómez-Rivada^1,2,3^, Christian Peters^4^, Joaquín Fernández-Irigoyen^5^, Enrique Santamaría^5^, M. Ángeles Rabadán^6^, Jordi Alberch^1,2,3,7^, Manuel J Rodríguez^1,2,3^, Daniel del Toro^1,2,3,7,*^ and Albert Giralt^1,2,3,7,*^

**^†^These authors contributed equally to this work.**

**^*^These senior authors contributed equally to this work and they are co-corresponding authors.**

**Author affiliations:**

1 Departament de Biomedicina, Facultat de Medicina, Institut de Neurociències, Universitat de Barcelona, 08036 Barcelona, Spain.

2 Institut d'Investigacions Biomèdiques August Pi i Sunyer (IDIBAPS), 08036 Barcelona, Spain.

3 Centro de Investigación Biomédica en Red sobre Enfermedades Neurodegenerativas (CIBERNED), 28031 Madrid, Spain.

4 Department of Molecules–Signaling–Development, Max-Planck Institute for Biological Intelligence, 82152 Martinsried, Germany.

5 Proteomics Platform, Navarrabiomed, Hospital Universitario de Navarra (HUN), Universidad Pública de Navarra UPNA, IdiSNA, 31008, Pamplona, Spain.

6 ZeClinics SL. and ZeNeuroid SL., Barcelona, Spain.

7 Production and Validation Centre of Advanced Therapies (Creatio), Faculty of Medicine and Health Science, University of Barcelona, 08036 Barcelona, Spain.

Correspondence to: Albert Giralt. ORCID: 0000-0001-5334-0963.

Full address: Department of Biomedical Sciences. Medical School, University of Barcelona. Casanova 143, 08036, Barcelona (Spain).

E-mail: [albertgiralt@ub.edu](mailto:albertgiralt@ub.edu)

Correspondence may also be addressed to: Daniel del Toro. ORCID: 0000-0002-7416-2155.

E-mail: [danieldeltoro@ub.edu](mailto:danieldeltoro@ub.edu)


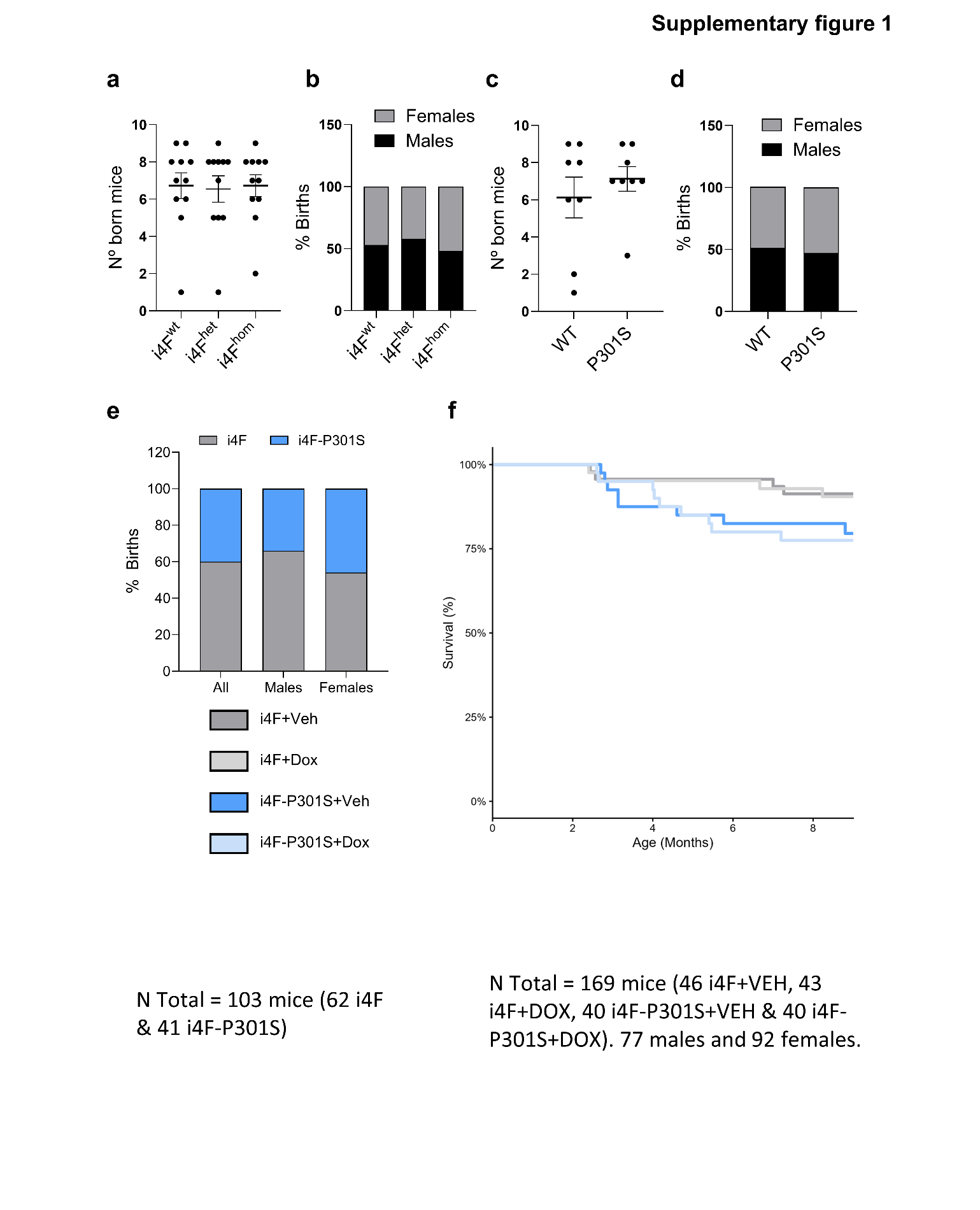


**Supplementary figure 1. Generation and viability of the i4F-P301S mouse line**. Mouse survival in terms of genotype and biological sex (**a**) as well as Mendelian distribution by sex (**b**) were both assessed in the i4F mouse line. Mouse survival in terms of genotype and biological sex (**c**) as well as Mendelian distribution by sex (**d**) were both assessed in the P301S mouse line. To generate the double heterozygous i4F-P301S mouse line we crossed homozygous i4F mice with heterozygous P301S mice. We then assessed Mendelian distribution by sex (**e**) and survival rates across lifespan (**f**) in this new generated line of double mutant the i4F-P301S mice. For **e** we monitored 103 mice and for **f** we monitored 169 mice.


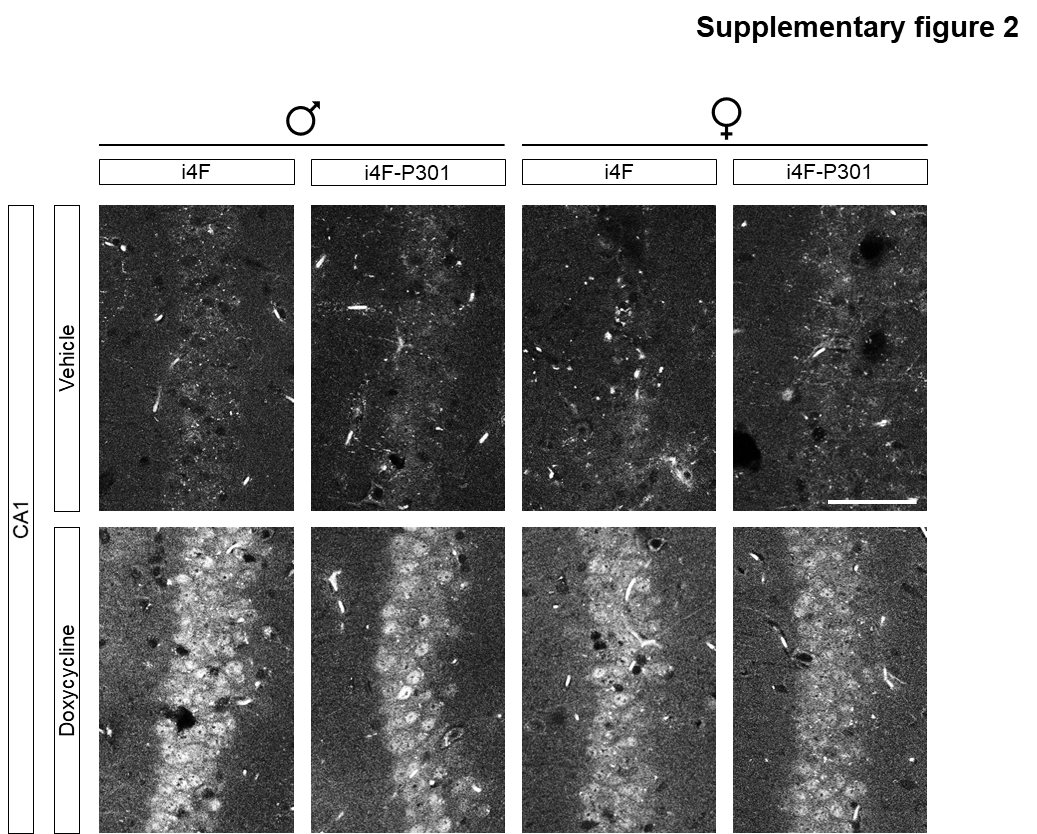


**Supplementary figure 2. KLF4 protein expression in reprogrammed mice**. Expression and distribution of KLF4 in i4F+Veh, i4F+Dox, i4F-P301S+Veh and i4F-P301S+Dox mice. Scale bar, 500 μm. Representative photomicrographs from dorsal CA1 showing KLF4 nuclear expression and localization in mice treated with Dox (lower row) but not in mice treated with Veh (upper row). Scale bar, 60 μm.


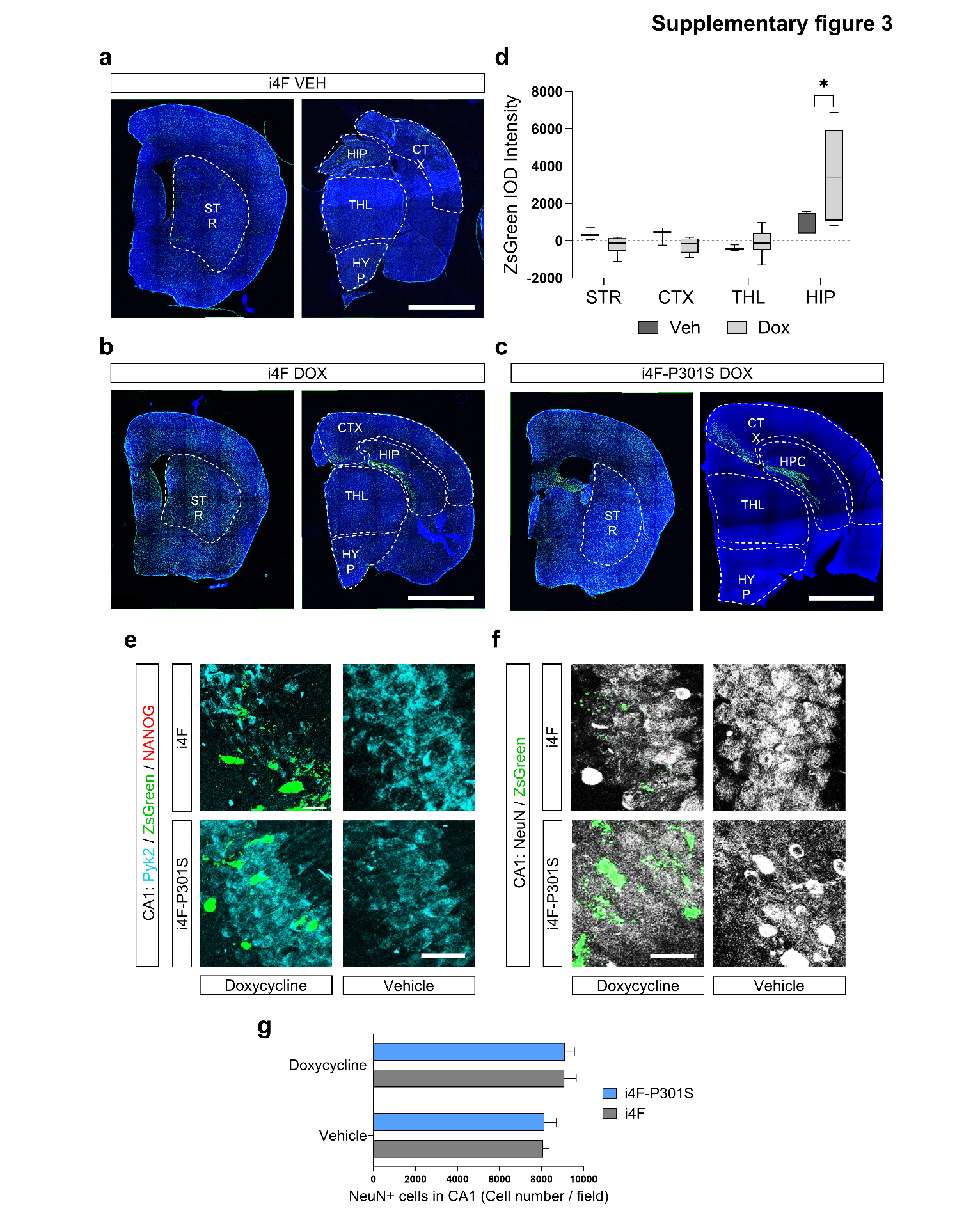


**Supplementary Figure 3. Regional specificity and safety of the intermittent and partial reprogramming strategy.** Expression and distribution of pAAV8[TetOn]-TRE>ZsGreen1-rev(SYN1>tTS:T2A:rtTA) were examined in the brains shown in **Fig. 2**, where the vector was stereotaxically delivered to the hippocampus and maintained for 6 months (from 12 to 36 weeks of age). Coronal mosaics of fixed brains from i4F-Veh, i4F-Dox, i4F-P301S+Veh, and i4F-P301S-Dox mice (n = 6 per group) were stained with DAPI (blue) and ZsGreen (green). No detectable recombination was observed in vehicle-treated groups (**a**). ZsGreen integrated optical density (IOD) was quantified in i4F-Dox (**b**, **d**) and i4F-P301S+Dox (**c**, **d**) mice across four brain regions (hippocampus (HIP), striatum (STR), cortex (CTX), and thalamus (THL)) to assess potential leakiness and vector spread following doxycycline administration. Scale bar: 2000 μm. High specificity of expression was confirmed in the hippocampus of doxycycline‑treated mice (Student's t-test, t = 2.363, df = 9, P = 0.042). (**e**) Immunostaining for NANOG (red), ZsGreen (green), and PYK2 in the hippocampus was performed to test whether transduced and partially reprogrammed neurons (ZsGreen+) exhibited signs of dedifferentiation (NANOG) or loss of mature neuronal identity (Pyk2). Transduced neurons retained mature neuronal features. Scale bar: 25 μm. (**f**) Immunostaining for NeuN (white) and ZsGreen (green) evaluated whether partial reprogramming affected neuronal maturity or density. Principal hippocampal neurons maintained NeuN expression. Scale bar: 25 μm. (**g**) NeuN-positive cell density from (**f**) was quantified in hippocampi from all four groups (n = 6–7 mice/group). No differences in neuronal density were detected (Two-way ANOVA, F_(1,21)_ = 0.4623; P = 0.5040).

**
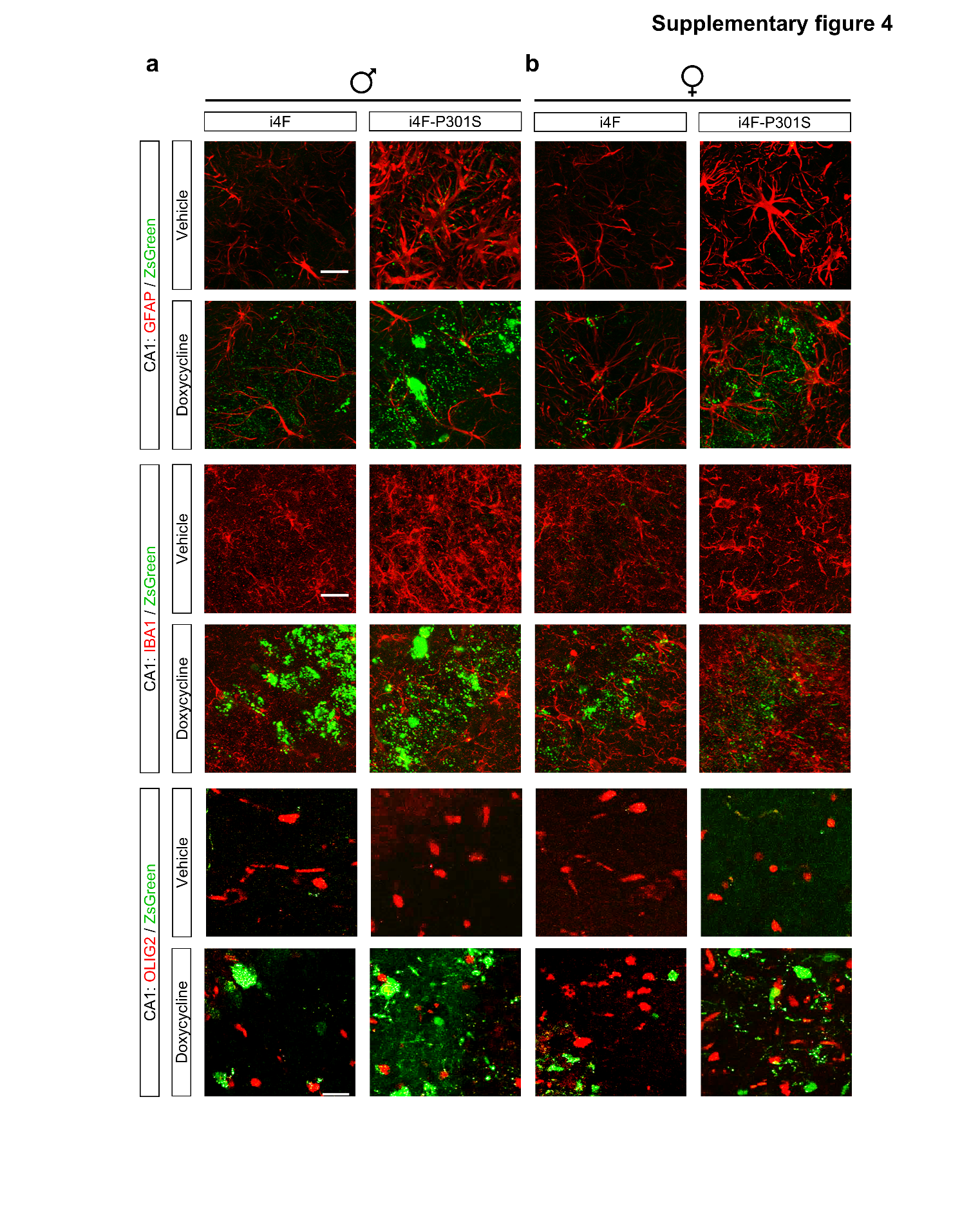
**

**Supplementary Figure 4. Hippocampal cellular specificity of the intermittent and partial reprogramming strategy.** Expression and distribution of pAAV8[TetOn]-TRE>ZsGreen1-rev(SYN1>tTS:T2A:rtTA) were examined at a cellular level in the hippocampus of i4F-Veh, i4F-Dox, i4F-P301S+Veh, and i4F-P301S-Dox mice after 5 months treatment (from 12 to 36 weeks of age) with Doxycycline or vehicle. Sections from males (**a**) and females (**b**) were stained for different cell types (in red) namely GFAP (astrocyte marker), IBA1 (microglial marker) and OLIG2 (oligodendroglia marker). ZsGreen (green) labeled transduced and reprogrammed cells. No detectable co-staining with ZsGreen and any of the aforementioned markers was observed. Scale bars: 25 μm.

**
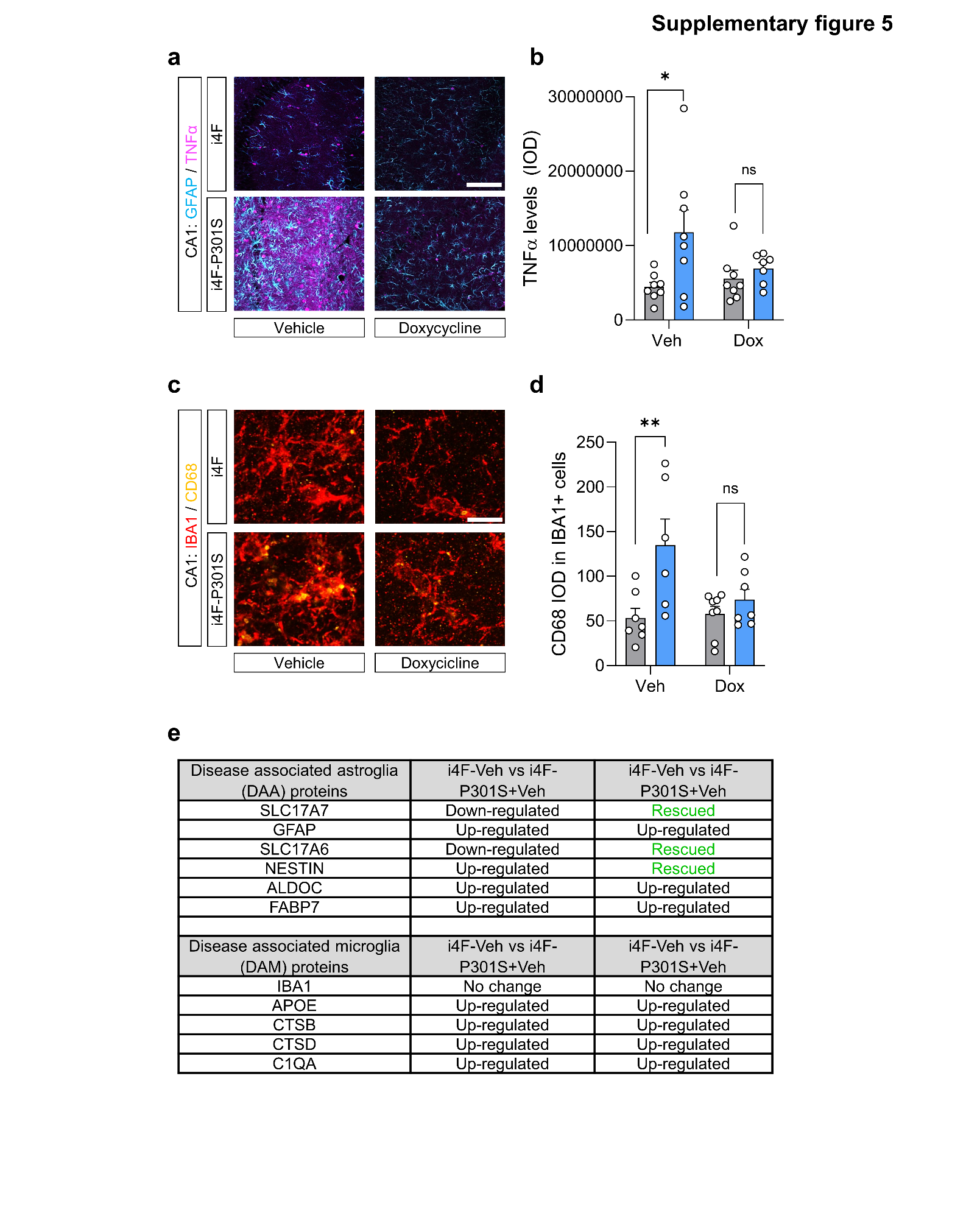
**

**Supplementary Figure 5. Characterization of astroglial and microglial function.** Hippocampal sections from the brains analyzed in **Figs. 3** and **4** were examined for additional potential alterations in glial cell states and functions. (**a**) Representative images showing GFAP (turquoise), an astrocytic marker, co‑stained with TNFα (pink), a pro‑inflammatory marker, in the CA1 region of i4F‑Veh, i4F‑Dox, i4F‑P301S+Veh, and i4F‑P301S‑Dox mice after 5 months of doxycycline or vehicle treatment (12–36 weeks of age). Scale bar: 60 μm. (**b**) Densitometric quantification of TNFα immunofluorescence from (**a**), expressed as integrated optical density (IOD). A significant effect was detected (Two‑way ANOVA, F_(1,27)_ = 6.363, P = 0.0179). A total of 7–8 mice per group (2 slices per mouse) were analyzed. (**c**) Representative images showing IBA1 (red), a microglial marker, co‑stained with CD68 (pink), an indicator of microglial phagocytic activity, in the CA1 region across the same four groups after 5 months of treatment. Scale bar: 15 μm. (d) Densitometric quantification of CD68 immunofluorescence normalized to the number of IBA1‑positive cells. A significant effect was observed (Two‑way ANOVA, F_(1,24)_ = 9.735; P = 0.0047). A total of 6–8 mice per group (2 slices per mouse) were analyzed. (**e**) Differentially expressed proteins associated with disease‑associated astroglia (DAA) and disease‑associated microglia (DAM) were identified from the mass spectrometry dataset (**Supplementary Table 1**). Only major proteins detected in our mass spectrometry analysis and classified as DAA or DAM are shown.


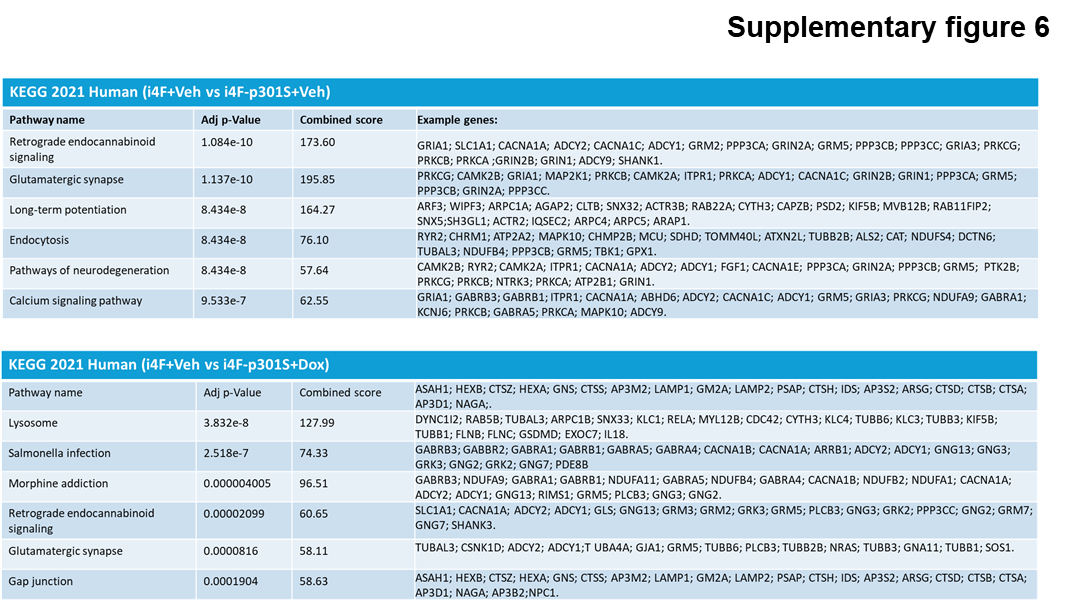


**Supplementary figure 6. Lists of synaptic differentially expressed proteins in reprogrammed mice.** Differentially expressed proteins using the KEGG 2021 Human database in two comparisons, i4F+Veh vs i4F-P301S+Veh (upper panel) and i4F+Veh vs i4F-P301S+Dox (lower panel)**.**
